# Causal effects of fatty acids on depression: Mendelian randomization study

**DOI:** 10.3389/fnut.2022.1010476

**Published:** 2022-12-06

**Authors:** Lingsi Zeng, Honggang Lv, Xubo Wang, Ranran Xue, Cong Zhou, Xia Liu, Hao Yu

**Affiliations:** ^1^Department of Psychiatry, Jining Medical University, Jining, Shandong, China; ^2^Department of Psychiatry, Shandong Daizhuang Hospital, Jining, Shandong, China; ^3^Department of Sleep Medicine, Shandong Daizhuang Hospital, Jining, Shandong, China

**Keywords:** depression, Mendelian randomization, fatty acids (FA), non-polyunsaturated fatty acids (non-PUFA), polyunsaturated fatty acids (PUFA), omega-3 fatty acids, omega-6 fatty acids

## Abstract

**Objectives:**

Fatty acids (FA) are widely believed to play a role in the pathophysiology of depression. However, the causal relationships between FA and depression remain elusive and warrant further research. We aimed to investigate the potential causal relationship between FA [saturated fatty acids (SFA), mono-unsaturated fatty acids (MUFA), and polyunsaturated fatty acids (PUFA)] and the risk of depression using Mendelian randomization (MR) analysis.

**Methods:**

We conducted a two-sample MR analysis using large-scale European-based genome-wide association studies (GWASs) summary data related to depression (*n* = 500,199 individuals) and FA [saturated fatty acids (SFA), mono-unsaturated fatty acids (MUFA), and polyunsaturated fatty acids (PUFA)] levels. MR analysis was performed using the Wald ratio and inverse variance-weighted (IVW) methods, and sensitivity analysis was conducted by the simple mode, weighted mode, weighted median method, and MR-Egger method.

**Results:**

We found the causal effects for the levels of oleic acid (OA; OR = 1.07, *p* = 5.72 × 10^–4^), adrenic acid (OR = 0.74, *p* = 1.01 × 10^–3^), α-linolenic acid (ALA; OR = 2.52, *p* = 1.01 × 10^–3^), eicosapentaenoic acid (EPA; OR = 0.84, *p* = 3.11 × 10^–3^) on depression risk, after Bonferroni correction. The sensitivity analyses indicated similar trends. No causal effect between the levels of SFA and depression risk was observed.

**Conclusion:**

Our study suggests that adrenic acid and EPA are protective against the risk of depression, while OA and ALA are potential risk factors for depression. Nonetheless, the underlying mechanisms that mediate the association between these FAs and depression risk should be investigated in further experiments.

## Introduction

Depression is a common and debilitating mental disorder that seriously affects psychosocial functioning and reduces the quality of life (1). Current evidence suggests that the disability-adjusted life-years (DALYs) due to depressive disorders have steadily increased over the past 30 years in the 10–24 and 25–49 age groups, impeding social and economic development and overwhelming the public health sector (2). Notwithstanding that the past decade has witnessed significant scientific advances, the mechanism and etiology of depression remain unclear, impeding the development of effective preventive treatment regimens and prognostic assessment (3–5). Diet represents a potentially modifiable factor that could be the focus of such preventative measures (6). The emergence of new area of research, “Nutritional Psychiatry” holds the promise of identifying which dietary components are indeed critical for mental health, including psychiatric disease. Moreover, it is essential to identify the populations, conditions, and dosages required for these nutritional interventions to exert preventative and therapeutic effects (7). The relationship between food and depression has received increasing attention (8).

Given that dietary factors may contribute to the onset of depression, a better understanding of this relationship can be harnessed to develop preventive measures for depression. Fatty acids (FA), which make up the bulk of fat in our bodies and food, are crucial for various physiological and pathological processes (9, 10). Common dietary FAs can be grouped as saturated fatty acids (SFA), mono-unsaturated fatty acids (MUFA), and polyunsaturated fatty acids (PUFA) based on the number of double bonds, type of double bonds, and chain length (11). SFAs have been linked to depressive symptoms, such as palmitic acid (PA) was positively associated with depressive symptoms, and arachidonic acid proportions showed negative association with depressive symptoms (12). MUFA fats were reported to have a negative effect on depressive symptoms (13). PUFAs, including long-chain omega-3 (mainly from fish), α-linolenic acid (ALA, a plant-based omega-3), and omega-6 (mainly from vegetable oils), participate in the synthesis, release, reuptake, and binding of neurotransmitters, as well as in the structure and function of the brain (9, 10, 12). Increased consumption of unsaturated FA, particularly PUFA, and decreased consumption of SFA have been found to lessen the risk of depression (14–17). These above findings demonstrate the importance of dietary fat content in research on the pathogenesis and treatment of depression. However, some studies have yielded inconsistent findings (18, 19). A previous randomized controlled trial (RCT) indicated an overall beneficial effect of omega-3 PUFAs on depression symptoms (20). Nevertheless, another large-scale RCT provides no support for the use of omega-3 PUFAs to prevent depression (21). Although it has been suggested that FA might contribute to the pathologic of depression risk, the potential causal relationship between FA and depression remains unclear.

Previous observational studies and meta-analyses have investigated the association between FA and depression risk (18, 20–22). However, observational studies cannot comprehensively evaluate causal links and exclude unreliable and biased factors. RCTs are the gold standard for studying causal links between exposure and outcome, which can be costly, impractical, or unethical (23). Furthermore, conventional observational studies can alter exposure-outcome relationships by confounding variables and reverse causal connections, restricting causal inference (24). Fortunately, in recent years, much emphasis has been placed on delineating the causal genetic variants and biological mechanisms based on genome-wide association studies (GWAS) methodology, generating a wide range of robust associations for various traits and disorders (25, 26). By utilizing genetic variations as a natural experiment, Mendelian randomization (MR) is a novel method to assess the apparent causal relationships between a potential exposure factor and the outcome (27). In addition, MR methods can overcome confounding and reverse causality association problems on causal inference (28–31). Moreover, the Two-sample MR method refers to the application of MR approach to summary-level GWAS results estimated in non-overlapping sets of individuals, but from the same ethnicity. In recent years, MR analysis has been used to examine the causative role of FA on ischemic stroke (32), frailty (33), Alzheimer’s disease (34), and schizophrenia (35). Nonetheless, little MR evidence is available on the relationship between FA and depression. To further analyze the influence of different types of FAs on depression, the causal link between FA and depression was assessed using MR. To conduct a comprehensive MR analysis between FA and depression, we selected 16 types of FAs, including SFAs [palmitic acid (PA), arachidic acid, and stearic acid (SA)], MUFAs [oleic acid (OA), and palmitoleic acid (POA)], omega-3 PUFAs [eicosapentaenoic acid (EPA), α-linolenic acid (ALA), docosapentaenoic acid (DPA), and docosahexaenoic acid (DHA)], omega-6 PUFAs [arachidonic acid (AA), adrenic acid, gamma–linolenic acid (GLA), Dihomo-gamma–linolenic acid (DGLA), and linoleic acid (LA)], total omega-3 PUFA and total omega-6 PUFA.

## Materials and methods

### Study design

**FIGURE 1:**
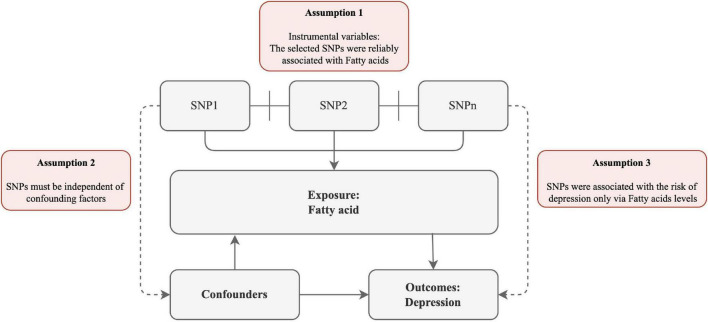
Schematic diagram of the assumptions underpinning a Mendelian randomization analysis of the association between plasma fatty acid (FA) levels and depression. We performed a two-sample MR study to examine the potential causal links between FA and the risk of depression. The MR method relies on the following assumptions: (1) the selected SNPs were reliably associated with FA; (2) SNPs must be independent of confounding factors; (3) SNPs were associated with the risk of depression only *via* FA levels.

To explore the potential causal links between FA and the risk of depression, we devised a two-sample MR study. The MR method relies on the following assumptions (Figure 1): (1) the selected SNPs were reliably associated with FA; (2) SNPs must be independent of confounding factors; (3) SNPs were associated with the risk of depression only *via* FA levels.

### Data sources

To ensure high power in the MR analysis and adequate reproducibility of results, our study collected publicly available GWAS summary data. First, we extracted SNPs associated with total omega-3 PUFA and total omega-6 PUFA at genome-wide significance level (*P* < 5 × 10^–8^) from the latest genome-wide association study (GWAS) of circulating PUFA, including 114,999 individuals of European ancestry (36). Next, we utilized several well-known meta-analysis genome-wide association results for the summary-level data of specific types of omega-3 and omega-6 PUFAs (37–40). Overall, we selected 52 SNPs as the genetic instruments for specific types of omega-3 PUFAs [docosapentaenoic acid (DPA, 3 SNPs), docosahexaenoic acid (DHA, 47 SNPs), α-linolenic acid (ALA, 1 SNP), eicosapentaenoic acid (EPA, 1 SNP)] and 64 SNPs as the genetic instruments for specific types of omega-6 PUFAs [arachidonic acid (AA, 2 SNPs), linoleic acid (LA, 57 SNPs), gamma-linolenic acid (GLA, 2 SNPs), dihomo-gamma-linolenic acid (DGLA, 2 SNPs), and adrenic acid (1 SNP)]. Moreover, summary statistics for non-PUFA were acquired from CHARGE (Cohorts for Heart and Aging Research in Genomic Epidemiology) (37). The summary statistics were generated from the European GWAS meta-analysis (*n* = 8,961 individuals for SFA or MUFA) (37). Moreover, we selected 5 SNPs as the genetic instruments for SFAs [PA (1 SNPs), arachidic acid (1 SNPs), and SA (3 SNPs)] and 5 SNPs as the genetic instruments for MUFAs [OA (1 SNP), and POA (4 SNPs)] (38). Finally, GWAS summary data on depression were obtained from the Psychiatric Genomics Consortium (PGC),^1^ including 246,363 cases and 561,190 controls (41). Considering the sample overlap between FA and depression GWAS, for total omega-3 and omega-6 PUFA levels, we extracted the SNP information from the meta-analysis of the 33 PGC cohorts, excluding UK Biobank, consisting of 135,548 cases and 344,901 controls (42). All GWAS summary data were mainly based on the GWAS of European cohorts. More details about these GWAS samples used in the present analysis are provided in Table 1.

**TABLE 1:** Information of included genome-wide association studies (GWAS) studies and consortia.

Phenotype	Ethnicity	*N*	References	Study
***Exposure: fatty acids***				
Total omega-3/omega-6 level	European	114,000	Borges et al. (36)	UK Biobank
omega-3 PUFAs (ALA, EPA, DPA, DHA)	European	8,866	Lemaitre et al. (37)	CHARGE consortium
omega-6 PUFAs (AA, GLA, DGLA, LA, Adrenic acid)	European	8,631	Guan et al. (39)	CHARGE consortium
Saturated fatty acids (PA, SA, Arachidic acid)	European	8,961	Wu et al. (38)	CHARGE consortium
Mono-unsaturated fatty acids (OA, POA)	European	8,961	Wu et al. (38)	CHARGE consortium
***Outcome: depression***				
Depression	European	807,553	Howard et al. (41)	PGC consortium
Depression	European	480,359	Wray et al. (42)	PGC consortium

PA, Palmitic acid; SA, Stearic acid; OA, Oleic acid; POA, Palmitoleic acid; AA, Arachidonic acid; GLA, Gamma-linolenic acid; DGLA, Dihomo-gamma-linolenic acid; LA, Linoleic acid; ALA, α-Linolenic acid; EPA, Eicosapentaenoic acid; DPA, Docosapentaenoic acid; DHA, Docosahexaenoic acid. We obtained public GWAS summary data from the website of CHARGE (https://www.chargeconsortium.com/) and PGC consortium (http://www.med.unc.edu/pgc/).

### Selection of genetic instrumental variants

We included the genome-wide significant single nucleotide polymorphisms (SNPs) (*p* < 5 × 10^–8^) as instrumental variables (IVs) (37–39, 41, 43). A more accurate effect size estimate with these variants was obtained as follows: genetic variants with high linkage disequilibrium (LD) (*R*^2^ > 0.001) were removed, and the distance between each variant was less than 10,000 kb. The SNPs were removed using the “clump” function implemented in the “TwoSampleMR” package (version 0.4.25). Furthermore, we removed palindromic variants with an ambiguous allele frequency from MR analyses (44).

### Statistical analysis

To investigate the association between FA and risk of depression, we applied the following MR methods, including Wald ratio, inverse variance weighted (IVW), MR-Egger, simple mode, weighted mode, and weighted median. If there is only one SNP as genetic instrumental variant, we performed MR analysis using the Wald ratio method (45). The Wald ratio method (i.e., the beta coefficient for the effect of the SNP on the outcome divided by the beta coefficient for the effect of the SNP on the exposure) was used to infer the causal association between exposure and the outcome containing only one SNP (45). For multiple SNPs as genetic instrumental variants, we conducted MR analysis by using the other methods, including IVW method, MR-Egger, weighted mode, simple mode, and weighted median. In MR analysis, the IVW method is the conventional method for summary statistics data and may be directly employed to calculate causal effect sizes without requiring individual-level data (46). Using regression analysis of the SNP-FA and the SNP-depression associations, we calculated the mean IVW estimates of SNP ratios (46). The weighted median method was used to estimate the impact effects, which enabled us to find the weighted empirical distribution function for all the selected SNPs. By utilizing the weighted median method, SNPs with stronger effects contribute more to causal estimates, and when fewer SNPs are effective tools, there is less bias toward estimating causal effects (47). The MR-Egger analysis was conducted assuming that the magnitude of the pleiotropic effects and SNP exposure effects are uncorrelated, allowing for a non-zero intercept in the regression model and unbalanced horizontal variability across all SNPs (48). MR-Egger regression performed a regression of the SNP-depression risk on the SNP-FA association. However, the MR-Egger method can provide unbiased estimates even if all selected SNPs are invalid (48). The results were presented as beta and their 95% confidence intervals (CI). The significant threshold after Bonferroni-adjustment for multiple tests was *P* < 0.003 (0.05/16 = 3.13E-3) since we conducted MR analyses of 16 types of FAs. To visualize the results for statistical analysis and the estimated effects for each SNP, we used the data analysis functionality of the MR-based platform to generate forest plots and scatter plots of the SNP-associated FA and depression risk (49). It is widely acknowledged that MR analysis results may be subject to significant heterogeneity due to differences in inclusion criteria, populations studied, and SNPs, thereby biasing causal effect inference. We conducted MR-Egger regression to assess the possible pleiotropy of SNPs used as IVs. The MR-Egger regression intercept implies the presence of directional horizontal pleiotropy drives the results from MR analyses (48). MR-Egger regression and IVW approaches were used to detect underlying heterogeneity of the association between different genetic variants. The significant heterogeneity was quantified with (*p*-value for Cochran’s Q statistic < 0.05). All analyses were performed using the TwoSampleMR package (version 0.5.6) in R (version 4.1.2) (49).

## Results

### Characteristics of single nucleotide polymorphisms associated with fatty acids as instrumental variables

After removing palindromic variants and SNPs with high LD (*R*^2^ > 0.001; distance < 1,000 kb), we identified SNPs associated with total omega-3 PUFA (*n* = 48), total omega-6 PUFA (*n* = 64), ALA (*n* = 1), EPA (*n* = 1), DPA (*n* = 3), DHA (*n* = 30), AA (*n* = 2), adrenic acid (*n* = 1), GLA (*n* = 2), DGLA (*n* = 2), LA (*n* = 44), POA (*n* = 4), OA (*n* = 1), SA (*n* = 2), arachidic acid (*n* = 1), and PA (*n* = 1) in the depression GWAS. These SNPs were not considered weak instrumental variables (*F*-statistic ≥ 10) and were eligible for MR analyses. Supplementary Table 1 provides details of the SNPs and their correlations to each type of FAs.

### Causal effects of omega-3 fatty acids on depression

**FIGURE 2:**
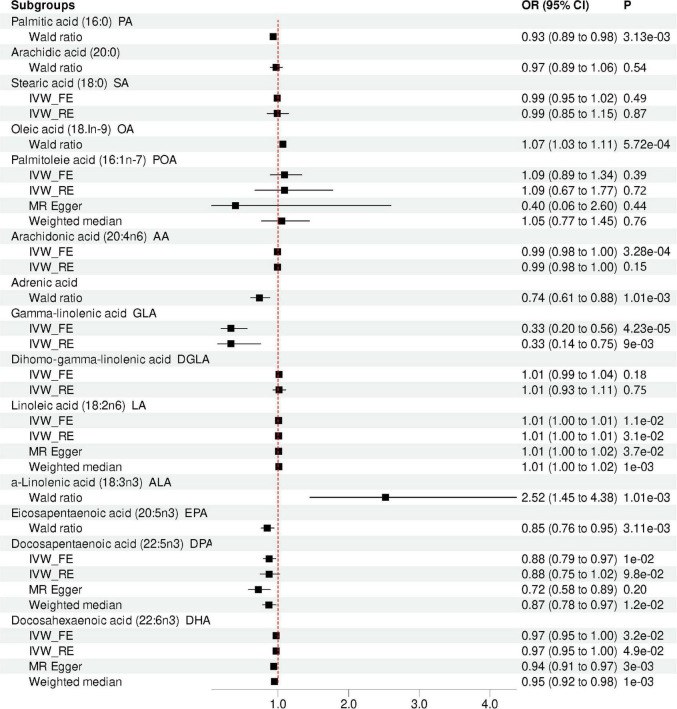
Mendelian randomization analysis showing the effects of different type of FA on depression risk. IVW_FE: fixed-effects inverse-variance weighted model; IVW_RE: random effect inverse variance weighted; CI, confidence interval; OR, odd ratio.

Our MR analysis indicated that the level of total omega-3 PUFA was not significantly associated with the risk of depression using the IVW method (OR = 0.96, 95% CI = 0.91 to 1.00, *p* = 0.07; Table 2 and Figure 2). Modified Q statistics indicated no notable heterogeneity (*p* = 0.73; Supplementary Table 2) across instrument SNP effects. Moreover, there was no horizontal pleiotropy by the MR-Egger intercept test (*p* = 0.73; Supplementary Table 2). The scatter plot, forest plot, funnel plot, and leave-one-out plot for the MR associations between total omega-3 PUFA and depression are provided in Supplementary Figure 1. Among the four types of omega-3 PUFAs, we detected a significant causal effect for ALA (OR = 2.52, 95% CI = 1.45 to 4.38, *p* = 1.01E-03) and EPA (OR = 0.84, 95% CI = 0.76 to 0.95, *p* = 3.11E-03) on depression risk (Table 2 and Figure 2) after Bonferroni correction. However, the levels of DPA (OR = 0.88, 95% CI = 0.79 to 0.97, *p* = 9.84E-03) and DHA (OR = 0.97, 95% CI = 0.95 to 1.00, *p* = 3.21E-02) showed no causal association with depression risk after Bonferroni correction. The forest plot, scatter plot, funnel plot, and leave-one-out plot for the MR associations between omega-3 PUFA, DPA, DHA, and depression risk are shown in Supplementary Figures 2, 3.

**TABLE 2:** Associations of plasma fatty acid levels with risk of depression in Mendelian randomization analyses (Wald ratio, multiplicative random effects inverse-variance weighted model fixed-effects inverse-variance weighted model).

FA subtype		Fatty acids	No of SNPs	Method	*P*	OR	95% CI	
Non-PUFAs								
	Saturated fatty acids	Palmitic acid (16:0) PA	1	Wald ratio	3.13E-03	0.93	0.886	0.976
		Arachidic acid (20:0)	1	Wald ratio	5.39E-01	0.973	0.891	1.062
		Stearic acid (18:0) SA	2	Inverse variance weighted (fixed effects)	4.88E-01	0.987	0.953	1.023
			2	Inverse variance weighted (multiplicative random effects)	8.72E-01	0.987	0.847	1.151
	Mono-unsaturated fatty acids	Oleic acid (18. In-9) OA	1	Wald ratio	5.72E-04	1.07	1.029	1.112
		Palmitoleic acid (16:1n-7) POA	4	Inverse variance weighted (fixed effects)	3.87E-01	1.093	0.893	1.339
			4	Inverse variance weighted (multiplicative random effects)	7.18E-01	1.093	0.674	1.775
			4	MR Egger	4.36E-01	0.396	0.06	2.6
			4	Simple median	6.89E-01	1.071	0.765	1.499
			4	Weighted median	7.55E-01	1.052	0.765	1.446
			4	Simple mode	9.87E-01	1.006	0.509	1.989
			4	Weighted mode	6.52E-01	0.835	0.411	1.697
PUFAS								
	n-6 PUFAs	Arachidonic acid (20:4n6) AA	2	Inverse variance weighted (fixed effects)	3.28E-04	0.991	0.985	0.996
			2	Inverse variance weighted (multiplicative random effects)	1.52E-01	0.991	0.978	1.004
		Adrenic acid	1	Wald ratio	1.01E-03	0.736	0.613	0.884
		Gamma-linolenic acid GLA	2	Inverse variance weighted (fixed effects)	4.23E-05	0.331	0.195	0.562
			2	Inverse variance weighted (multiplicative random effects)	8.53E-03	0.331	0.145	0.754
		Dihomo-gamma-linolenic acid DGLA	2	Inverse variance weighted (fixed effects)	1.76E-01	1.015	0.993	1.037
			2	Inverse variance weighted (multiplicative random effects)	7.46E-01	1.015	0.928	1.109
		Linoleic acid (18:2n6) LA	40	Inverse variance weighted (fixed effects)	1.05E-02	1.008	1.002	1.013
			40	Inverse variance weighted (multiplicative random effects)	3.13E-02	1.008	1.001	1.014
			40	MR Egger	3.70E-02	1.008	1.001	1.016
			40	Simple median	8.87E-01	1.003	0.965	1.043
			40	Weighted median	9.54E-04	1.01	1.004	1.016
			40	Simple mode	7.50E-01	0.991	0.936	1.049
			40	Weighted mode	5.74E-03	1.009	1.003	1.015
	n-3 PUFAs	α-Linolenic acid (18:3n3) ALA	1	Wald ratio	1.01E-03	2.522	1.453	4.377
		Eicosapentaenoic acid (20:5n3) EPA	1	Wald ratio	3.11E-03	0.849	0.761	0.946
		Docosapentaenoic acid (22:5n3) DPA	3	Inverse variance weighted (fixed effects)	9.84E-03	0.876	0.792	0.969
			3	Inverse variance weighted (multiplicative random effects)	9.84E-02	0.876	0.748	1.025
			3	MR Egger	2.02E-01	0.72	0.583	0.89
			3	Simple median	8.90E-01	1.015	0.821	1.255
			3	Weighted median	1.25E-02	0.871	0.782	0.971
			3	Simple mode	8.60E-01	1.027	0.789	1.337
			3	Weighted mode	1.03E-01	0.845	0.753	0.948
		Docosahexaenoic acid (22:6n3) DHA	30	Inverse variance weighted (fixed effects)	3.21E-02	0.973	0.949	0.998
			30	Inverse variance weighted (multiplicative random effects)	4.89E-02	0.973	0.947	1
			30	MR Egger	2.56E-03	0.94	0.907	0.975
			30	Simple median	7.50E-01	0.991	0.938	1.048
			30	Weighted median	1.19E-03	0.949	0.919	0.979
			30	Simple mode	3.96E-01	0.966	0.893	1.045
			30	Weighted mode	4.34E-03	0.954	0.926	0.983
	n-3 PUFAs	Omega-3 FAs	37	Inverse variance weighted	7.00E-02	0.956	0.911	1.004
			37	MR Egger	1.50E-01	0.947	0.881	1.018
			37	Weighted median	1.21E-01	0.945	0.879	1.015
	n-6 PUFAs	Omega-6 FAs	47	Inverse variance weighted	8.38E-01	1.007	0.944	1.073
			47	MR Egger	5.80E-02	1.137	0.999	1.294
			47	Weighted median	8.47E-01	1.009	0.924	1.102

CI, confidence interval; OR, odd ratio.

### Causal effects of omega-6 fatty acids on depression

Our results suggested that the level of total omega-6 PUFA was not significantly associated with depression using the IVW method (OR = 1.00, 95% CI = 0.94 to 1.07, *p* = 0.84; Table 2 and Figure 2). Cochran’s *Q* of IVW analysis revealed no significant heterogeneity among SNPs (*p* = 0.73; Supplementary Table 2). Additionally, MR-Egger regression analysis showed no horizontal pleiotropy among SNPs between omega-6 FA and depression (*p* = 0.73; Supplementary Table 2). Supplementary Figure 4 displays scatter plots of the SNP-omega-6 FA and SNP-depression association estimates with five different MR methods, which visualized the causal effect estimates of each SNP on depression. In the funnel plot (Supplementary Figure 5), the causal effect point exhibited a symmetric distribution, indicating that a single SNP as IV was less likely to bias the cause. Among the five specific types of omega-6 PUFAs, genetically predicted level of AA (OR = 0.99, 95% CI = 0.99 to 1.00, *p* = 3.28E-04), adrenic acid (OR = 0.74; 95% CI = 0.61 to 0.88; *p* = 1.01E-03), and GLA (OR = 0.33, 95% CI = 0.20 to 0.56, *p* = 4.23E-05) were associated with depression, after Bonferroni correction. However, AA and GLA exhibited significant heterogeneity in the IVW analyses and was not significantly associated with the depression corrected by the multiplicative random-effects IVW method AA (OR = 0.99, 95% CI = 0.98 to 1.00, *p* = 0.15) and GLA (OR = 0.33, 95% CI = 0.15 to 0.75, *p* = 8.53E-03). Genetic predictions demonstrated no significant association between DGLA (OR = 1.02, 95% CI = 0.99 to 1.04, *p* = 0.18), LA (OR = 1.01, 95% CI = 1.00 to 1.01, *p* = 0.01) and depression after Bonferroni correction.

### Causal effects of saturated fatty acids on depression

Genetic predictions showed an association between PA levels and depression (OR = 0.93; 95% CI = 0.89 to 0.98; *p* = 3.13E-03), which was no longer observed after Bonferroni correction. Sensitivity analysis based on MR Egger and weighted median methods yielded similar results for the causal effects of PA levels on depression (Table 2 and Figure 2). Heterogeneity and horizontal pleiotropy analyses were not conducted since only one SNP was left. However, after the Bonferroni correction, no association was found between the levels of SA (OR = 0.99, 95%CI: 0.95 to 1.02, *p* = 0.49), Arachidic acid (OR = 0.97, 95%CI: 0.89 to 1.06, *p* = 0.54) and depression. The scatter plot, forest plot, funnel plot, and leave-one-out plot for the MR associations between SA and depression are provided in Supplementary Figure 5.

### Causal effects of mono-unsaturated fatty acids on depression

There was one single SNP associated with OA conducted using Wald ratio MR analyses were statistically associated with depression (OR = 1.07, 95% CI = 1.03 to 1.11, *p* = 5.72E-04), surviving after Bonferroni correction. However, after the Bonferroni correction, no association was found between the levels of POA and depression (OR = 1.09, 95% CI = 0.89 to 1.34, *p* = 0.39). The scatter plot, forest plot, funnel plot, and leave-one-out plot for the MR associations between POA and depression are provided in Supplementary Figure 6.

## Discussion

In this work, the MR approach was used to obtain accurate findings regarding the influence of FA on depression. We found that higher levels of adrenic acid and EPA were associated with a decreased risk of depression. In contrast, our MR results suggested that higher levels of OA and ALA could increase the risk of depression. Most importantly, the present investigation found no causal relationship between other omega-3 PUFAs (DHA and DPA), omega-6 PUFAs (AA, GLA, DGLA, and LA), MUFA (POA), and SFA (arachidic acid, PA, and SA) and depression risk. Our study was based on GWAS summary data and provided novel insights into the potential roles of specific FAs on depression risk for follow-up studies.

Our results indicated that elevated ALA levels were associated with increased depression risk, whereas higher EPA levels were associated with decreased depression risk. ALA is the precursor for long-chain PUFAs, including EPA and DHA (50), which are essential components of neuronal membrane phospholipids and regulate the physical and physiological features of neuronal membranes (51). Although the conversion efficiency is reportedly moderate, enough ALA consumption from flaxseed oil can dramatically increase the amounts of neuronal long-chain omega-3 PUFAs in rats (52). It has been established that neurotransmitter signaling in the GABAergic, serotonergic, or dopaminergic systems might be related to the neurobehavioral effects of ALA on depression risk (53). Our result for the causal relationship between EPA and depression is consistent with the earlier MR study (54). Growing evidence implicated that EPA plays a crucial role in the development and nervous system repair of synapse and nerve growth cones which are the basis of the neurodevelopmental hypothesis in depression (55–60). However, previous observational and clinical trial data on the association of omega-3 PUFA with depression have yielded conflicting results. A meta-analysis of observational studies suggested that marine omega-3 PUFA may reduce the risk of depression and promote a favorable mood (18). A cross-sectional observational study found no evidence for associations between DHA and omega-3 index and depression (19). The discrepancy among these observational studies may be explained by residual confounding. Additionally, the effect of supplementation with omega-3 PUFA on the risk of depression has been extensively studied over the years (20–22). For example, a meta-analysis of double-blind randomized placebo-controlled trials showed an overall beneficial effect of omega-3 PUFAs with EPA ≥ 60% at a dosage of ≤1 g/d on depression symptoms, whereas DHA-pure and DHA-major formulations did not yield such benefits (20). However, a large-scale randomized clinical trial involving 18,353 adults aged 50 years or older found no evidence supporting the use of omega-3 supplements in adults to prevent depression (21). Therefore, we hypothesized that omega-3 PUFA supplementation might not be beneficial for the prevention of depression but might be beneficial (specifically EPA) for the treatment of MDD. The confounding factors may be responsible for the inconsistent outcomes. Given that MR techniques might overcome confounding variables and offer the causal effects of FA on depression, we hypothesized that increasing levels of ALA might increase the risk of depression.

Our MR study found no association between total omega-6 PUFA and depression, showing consistent results compared with previous observational studies (61, 62). In a previous cross-sectional observational study, no association was found between omega-6 PUFA levels and depression (61). Meanwhile, observational research revealed that circulating omega-6 PUFA levels were not associated with depression relapse risk (62). As for the specific type of omega-6 PUFAs, the current investigation revealed that the higher level of adrenic acid was associated with a reduced risk of depression. Adrenic acid, the immediate precursor derived from arachidonic acid, is necessary for neuronal development and enrichment of myelinic lipids (63, 64). Adrenic acid is metabolized by cytochrome P450s to a group of epoxy fatty acids and epoxyeicosatrienoic acids, which are the vital lipid mediators playing key role in exerting analgesia and reducing endoplasmic reticulum stress (65). Furthermore, functional studies showed that adrenic acid potently inhibited the formation of the chemoattractant leukotriene B4 (LTB4), which has been proposed as a contributor for chronic neuroinflammatory effects (66–68). Given that, we could pay more attention to the role of the specific type of omega-6 PUFAs in depression, such as adrenic acid. Our findings might provide novel insights into the link between omega-6 PUFA and depression, given the paucity of existing research on the associations between omega-6 PUFA levels and depression.

Our MR analysis found a significant causal effect between specific types of MUFAs and the risk of depression. Little evidence is available on the association between MUFA and the risk of depression. The consumption of OA was positively related to depressive symptoms, according to a cross-sectional study involving 2,793 perimenopausal women (69). Mechanically, neural differentiation depends on OA production (70). Moreover, it has been shown that the composition of FA in the hippocampus and brain of rats with cognitive impairment is altered by OA (71). Hamilton and Fernandes (72) found that co-infusion with OA reduced the abundance of proliferating neural stem cells (NSCs) and suppressed the activation of NSCs in mice models. Therefore, OA might be involved in depression by affecting neural functions. However, the relationships between OA and depression remain largely unclear, warranting further experiments.

Importantly, in the present study, we adopted MR analyses which can avoid reverse causality induced by intrinsic confounders in conventional observational research. Moreover, due to the relatively large sample size, our MR study had more statistical power, which made the findings more accurate. However, some limitations must be considered when interpreting our findings. Our current MR investigation used GWAS summary data, and the study populations were predominantly European. The applicability of the study’s findings to other populations remains unclear, emphasizing the need for more studies. Besides, the original GWAS data of depression and characteristics of subjects could not be retrieved directly. Consequently, no subgroup analysis according to characteristics of subjects (sex, geography, and so on) was conducted. Moreover, the MR approach could only provide a preliminary indication of the causative link between FA and depression. The biological mechanism between FA and depression would be comprehensively investigated the further experiments.

## Conclusion

Overall, our MR analyses provide evidence that ALA and OA levels increase the risk of depression, whereas EPA and adrenic acid levels decrease the risk of depression. Nonetheless, more in-depth work is still needed to elucidate the underlying mechanisms that mediate the relationship between ALA, OA, EPA, and adrenic acid levels and depression.

## Data availability statement

The original contributions presented in this study are included in the article/Supplementary material, further inquiries can be directed to the corresponding authors.

## Author contributions

HL, LZ, and HY designed the study, contributed to analysis and interpretation of data, and wrote the first draft of the manuscript. HL, LZ, XW, XL, RX, CZ, and HY did the statistical analyses and prepared the tables and figures. HY provided further data interpretation. All authors contributed to drafting the work or revising it critically for important intellectual content and made substantial contributions to the concept and design of the study and acquisition, analysis, and interpretation of data.

## Footnotes

1
http://www.med.unc.edu/pgc/
