# Supplementary material for: Causal effects of fatty acids on depression: Mendelian randomization study
Source: Front Nutr. 2022 Dec 6;9:1010476. doi: 10.3389/fnut.2022.1010476 (PMC9763462; doi:10.3389/fnut.2022.1010476)
Supplement: Supplementary file 1 [file Data_Sheet_1.docx]

**Supplemental Material:**

**Causal Effects of Fatty Acids on Depression: Mendelian Randomization Study**

# Supplementary Figures:

Figure S1. Mendelian randomization (MR) sensitivity plots of the causal effect of Omega-3 Fatty Acids levels on Depression. (A, B, C, D) ---------------------------------------------------[2](#FigureS1)

Figure S2. Mendelian randomization (MR) sensitivity plots of the causal effect of Docosapentaenoic acid (DPA) levels on Depression. (A, B, C, D)------------------------------[6](#FigureS2)

Figure S3. Mendelian randomization (MR) sensitivity plots of the causal effect of Docosahexaenoic acid (DHA) levels on Depression. (A, B, C, D)------------------------------[10](#FigureS4)

Figure S4. Mendelian randomization (MR) sensitivity plots of the causal effect of Omega-6 Fatty Acids levels on Depression. (A, B, C, D)----------------------------------------------------[14](#FigureS5)

Figure S5. Mendelian randomization (MR) sensitivity plots of the causal effect of Stearic acid (SA) levels on Depression. (A, B, C, D)-------------------------------------------------------[18](#FigureS6)

Figure S6. Mendelian randomization (MR) sensitivity plots of the causal effect of Palmitoleic acid (POA) levels on Depression. (A, B, C, D)---------------------------------------[22](#FigureS7)

# Supplementary Tables:

Table S1. Instrumental variables associated with various subtypes of fatty acids level were used in the present analysis. ---------------------------------------------------------------------------------[26](#TableS1)

Table S2. The heterogeneity and pleiotropy test between specific FAs and depression. ------[34](#TableS2)

**Figure S1. Mendelian randomization (MR) sensitivity plots of the causal effect of total Omega-3 Fatty Acids levels on Depression.**

**A. Scatter plot.** Scatter plots of genetic association with total Omega-3 Fatty Acids levels on Depression showing comparison of the causal estimates from the various Mendelian randomization methods.

**
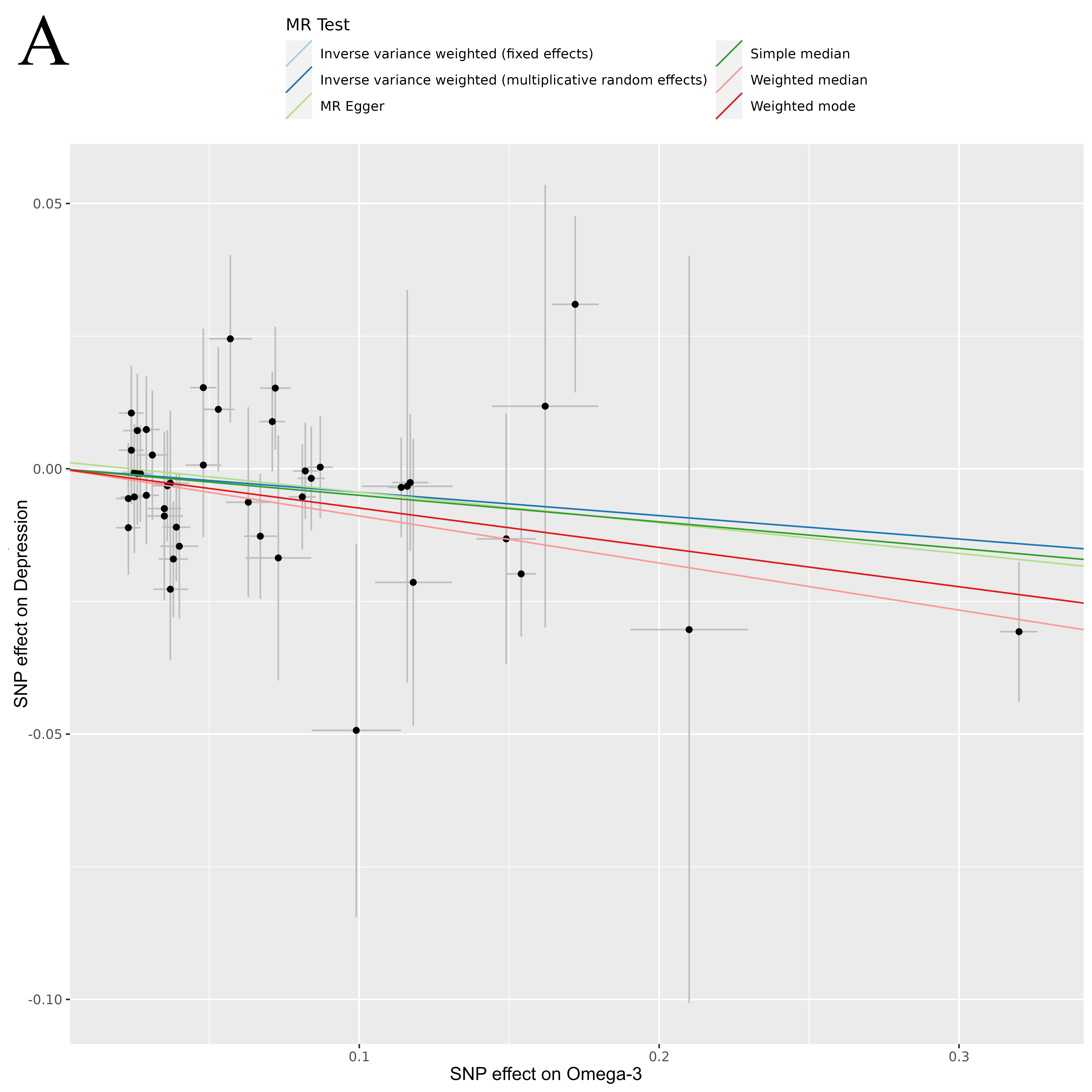
**

**B. Funnel plot.** Funnel plot of causal association between total Omega-3 Fatty Acids levels and Depression.


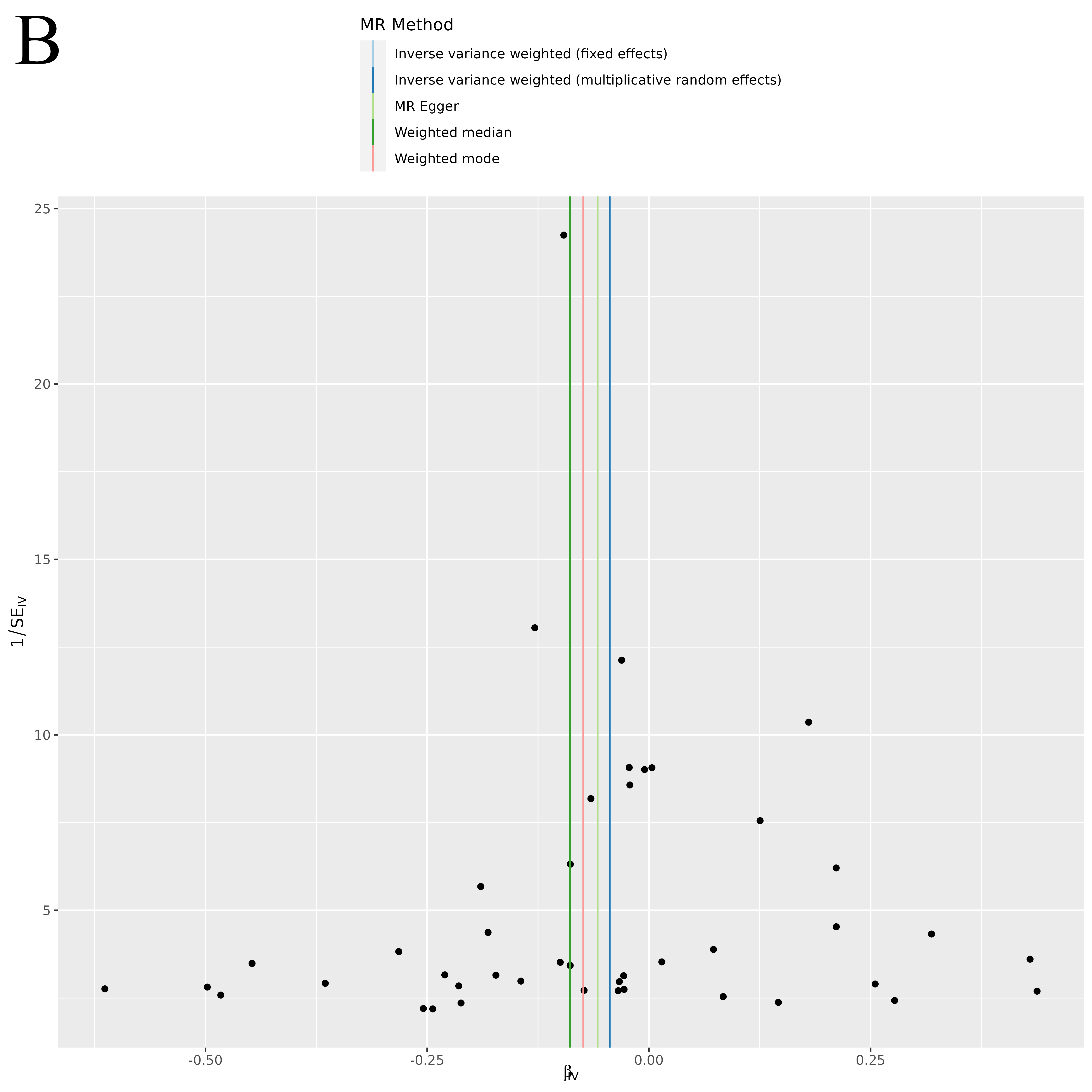


**C. Forest plot.** Forest plot of variant specific inverse variance estimates for causal association between total Omega-3 Fatty Acids levels and Depression.

**
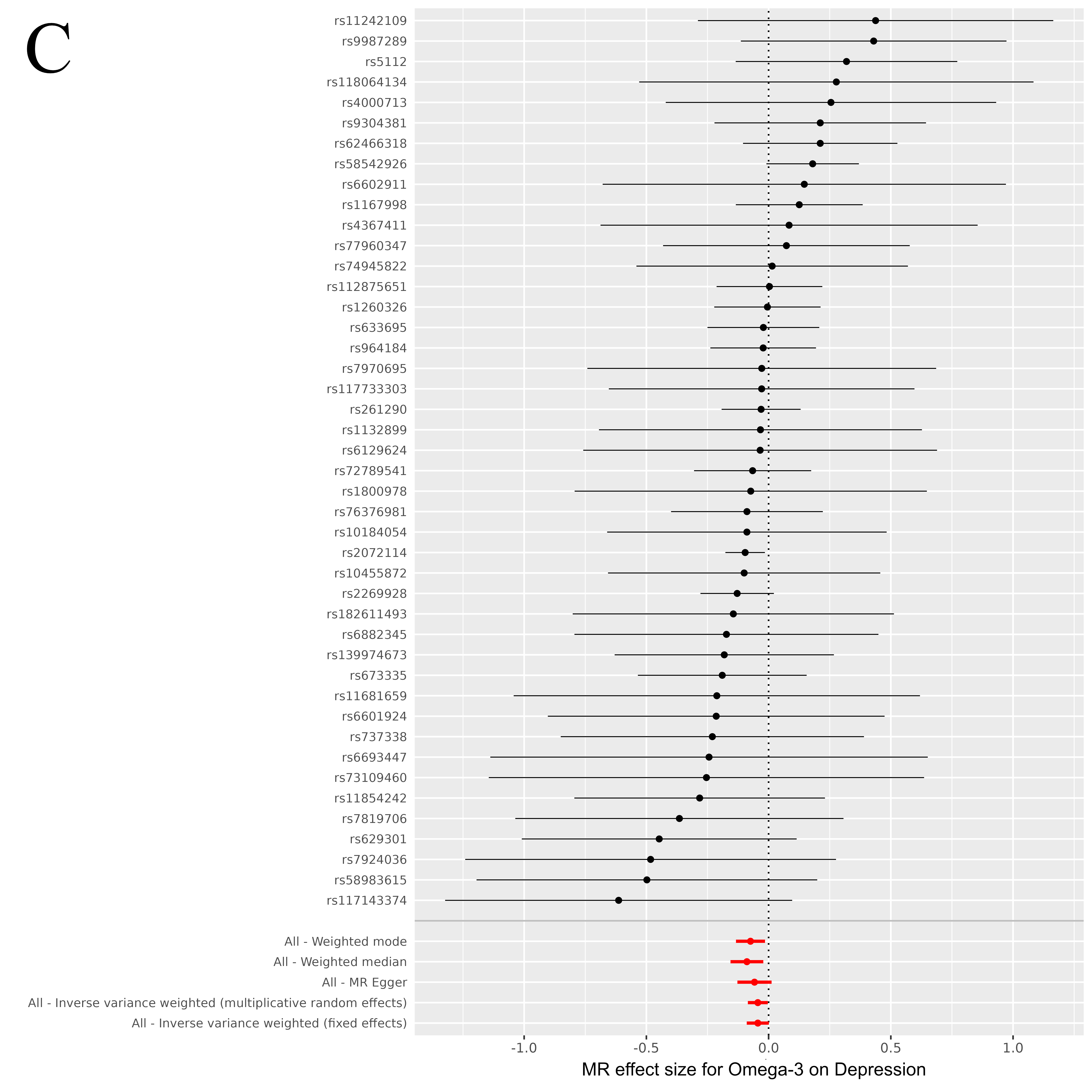
**

**D. Leave-one-out plot.** Leave-one-out plot to assess if a single variant is driving the association between total Omega-3 Fatty Acids levels and Depression.

**
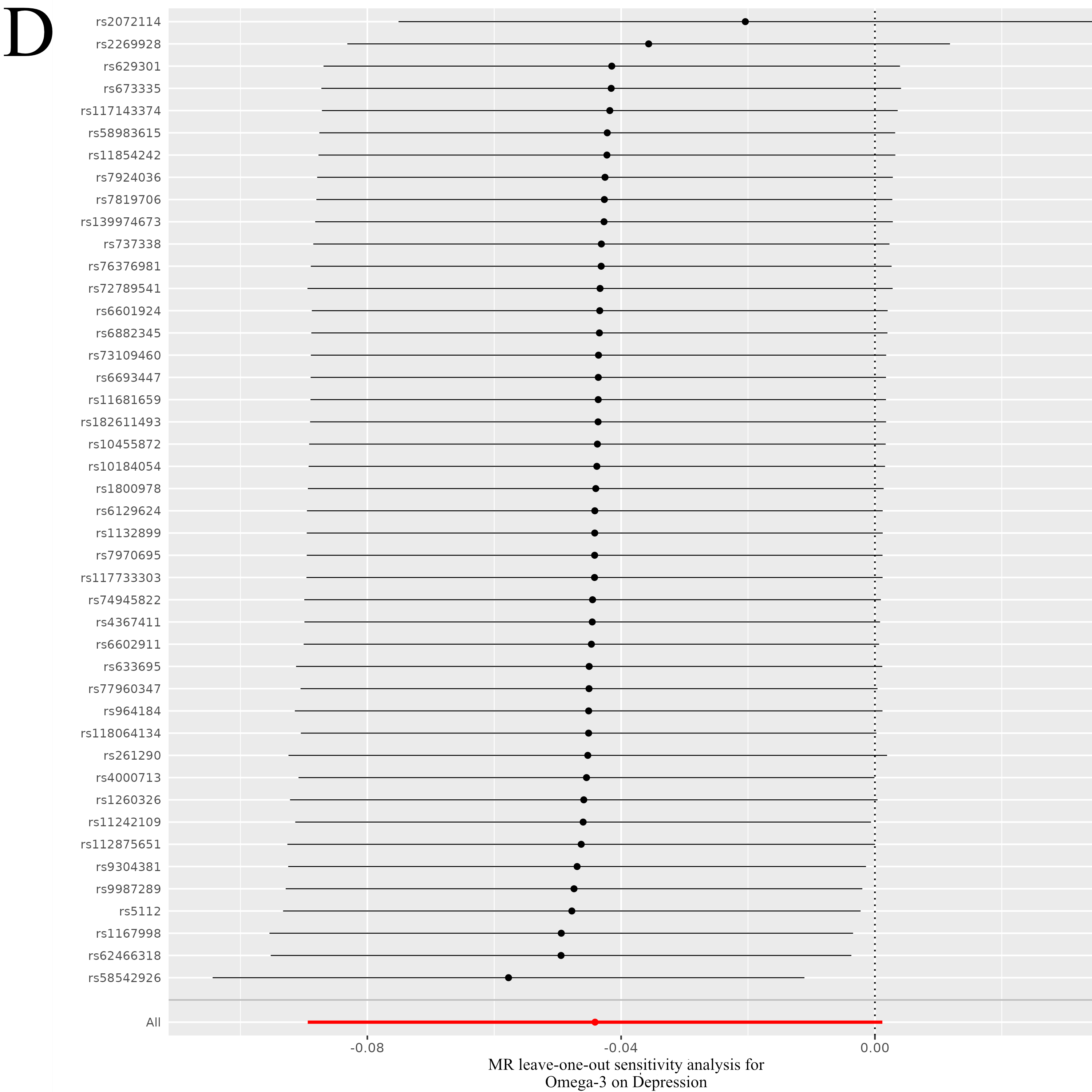
**

**Figure S2. Mendelian randomization (MR) sensitivity plots of the causal effect of Docosapentaenoic acid (DPA) levels on Depression.**

**A. Scatter plot.** Scatter plots of genetic association with Docosapentaenoic acid (DPA) levels on Depression showing comparison of the causal estimates from the various Mendelian randomization methods.

**
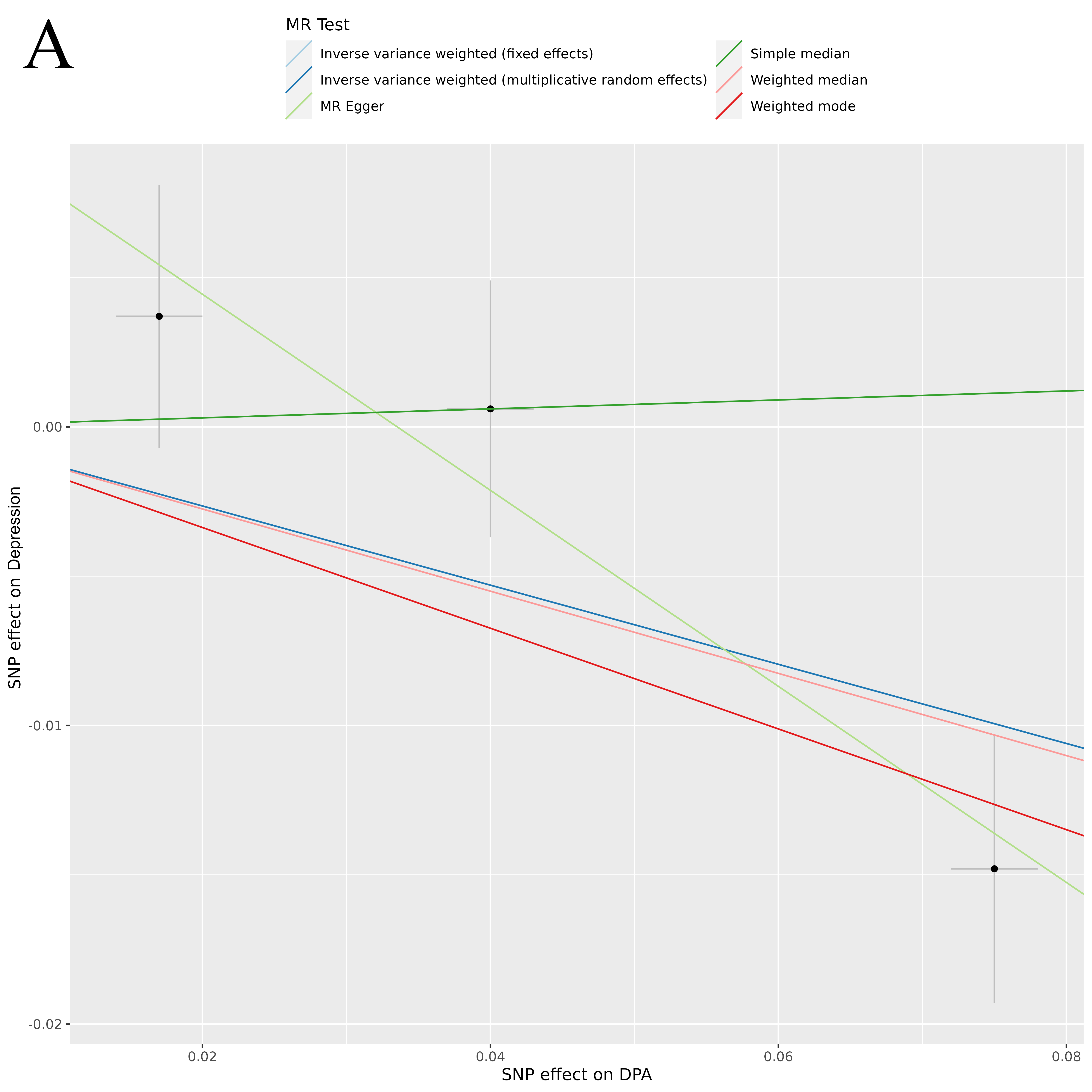
**

**B. Funnel plot.** Funnel plot of causal association between Docosapentaenoic acid (DPA) levels and Depression.**
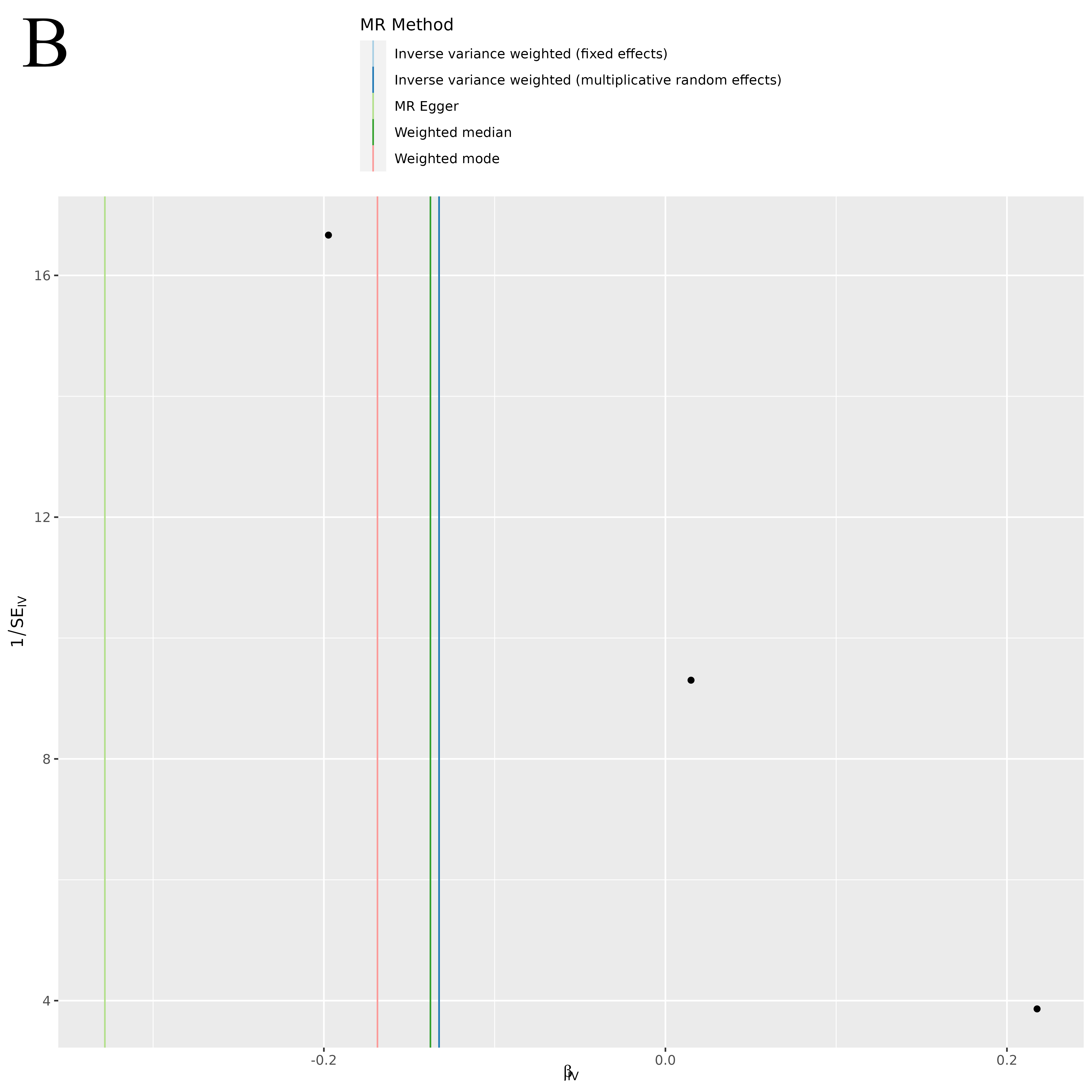
**

**C. Forest plot.** Forest plot of variant specific inverse variance estimates for causal association between Docosapentaenoic acid (DPA) levels and Depression.

**
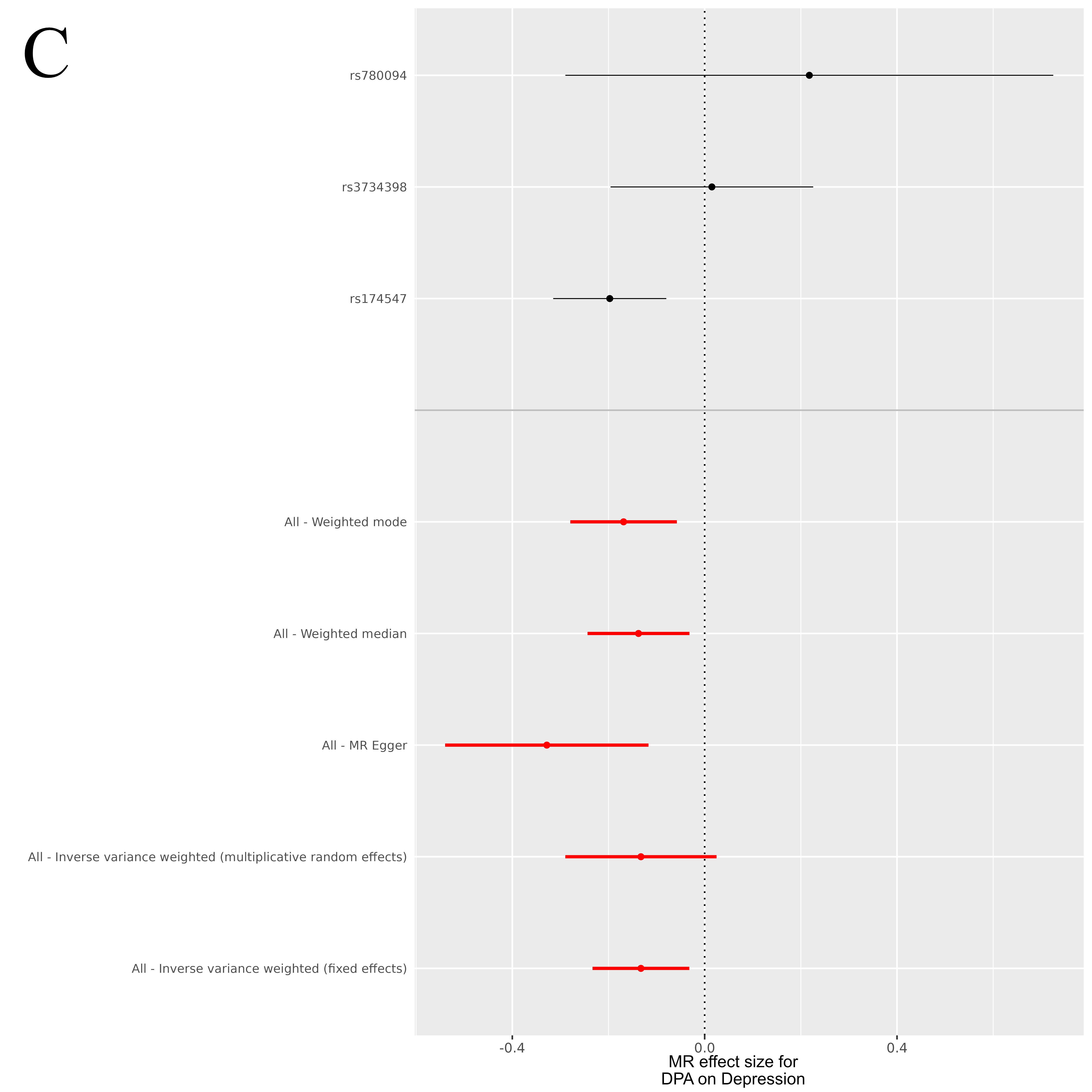
**

**D. Leave-one-out plot.** Leave-one-out plot to assess if a single variant is driving the association between Docosapentaenoic acid (DPA) levels and Depression.

**
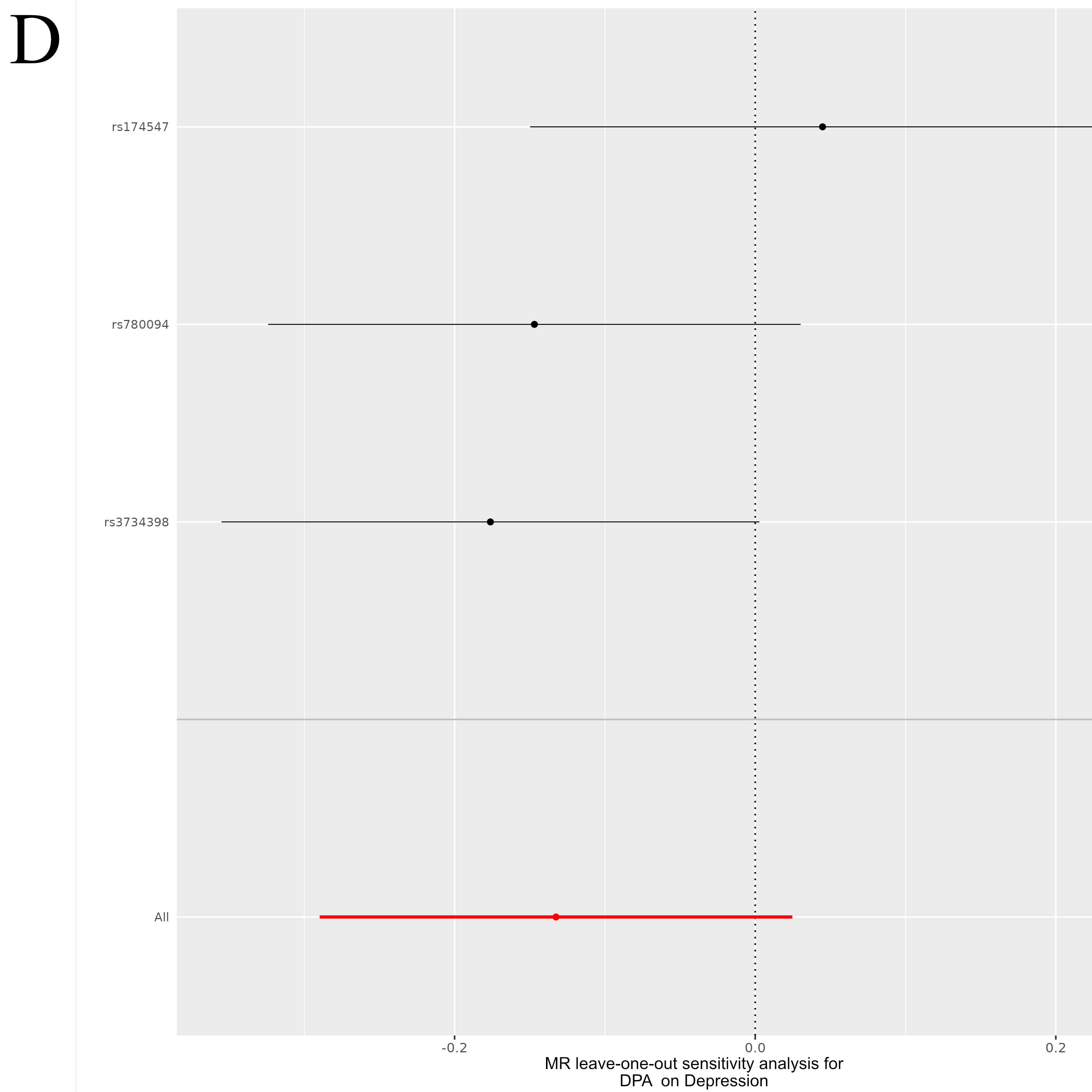
**

**Figure S3. Mendelian randomization (MR) sensitivity plots of the causal effect of Docosahexaenoic acid (DHA) levels on Depression.**

**A. Scatter plot.** Scatter plots of genetic association with Docosahexaenoic acid (DHA) levels on Depression showing comparison of the causal estimates from the various Mendelian randomization methods.

**
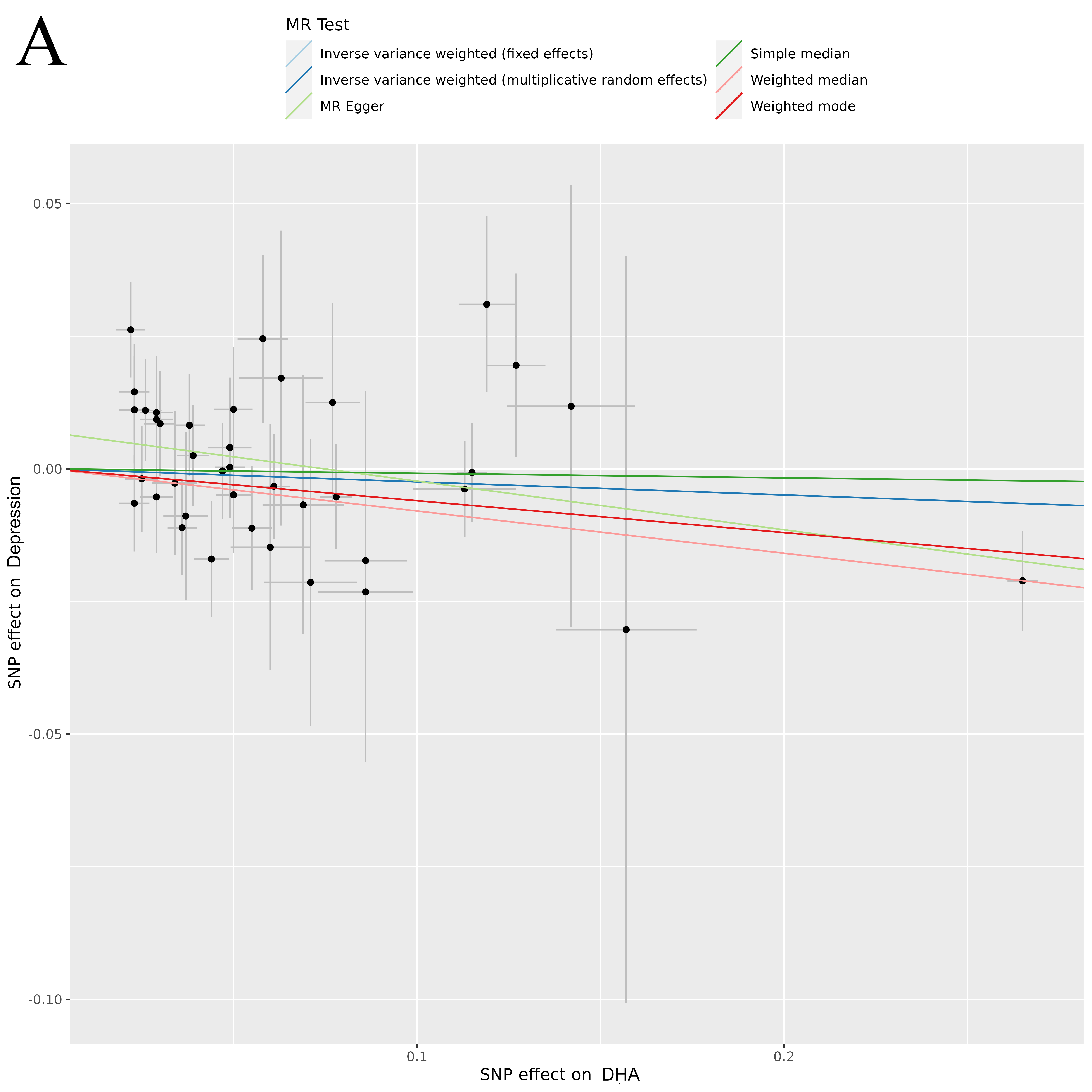
**

**B. Funnel plot.** Funnel plot of causal association between Docosahexaenoic acid (DHA) levels and Depression.

**
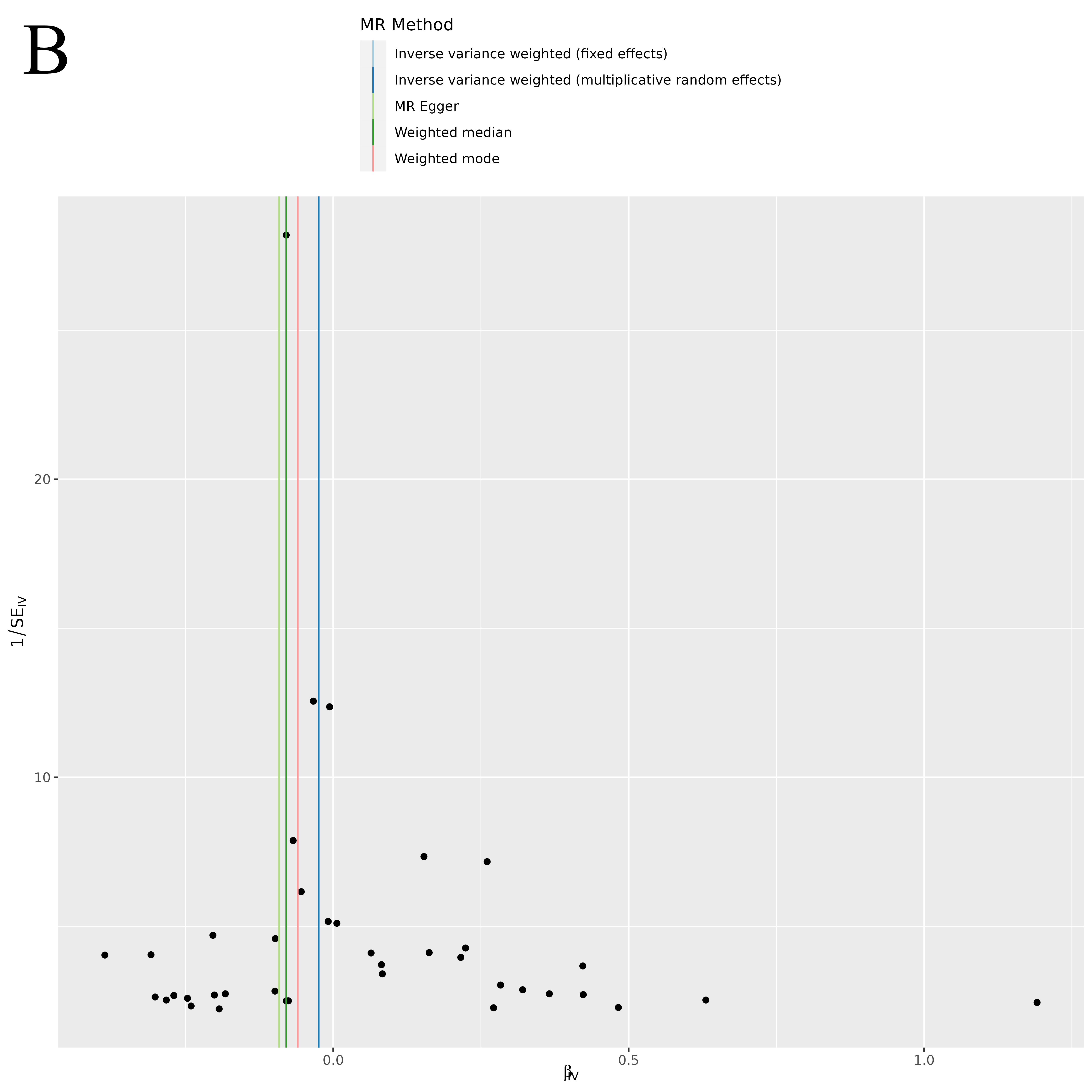
**

**C. Forest plot.** Forest plot of variant specific inverse variance estimates for causal association between Docosahexaenoic acid (DHA) levels and Depression.

**
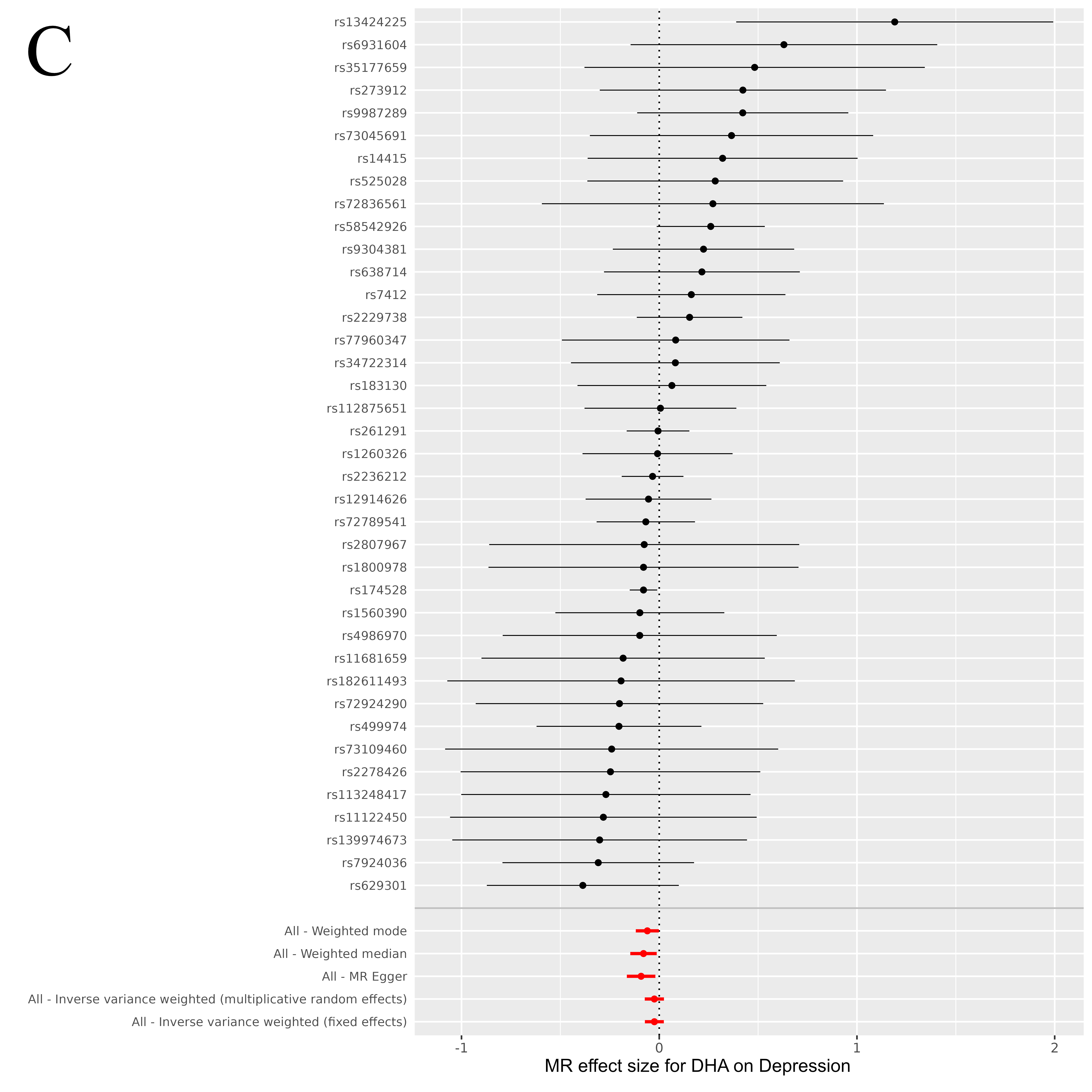
**

**D. Leave-one-out plot.** Leave-one-out plot to assess if a single variant is driving the association between Docosahexaenoic acid (DHA) levels and Depression.

**
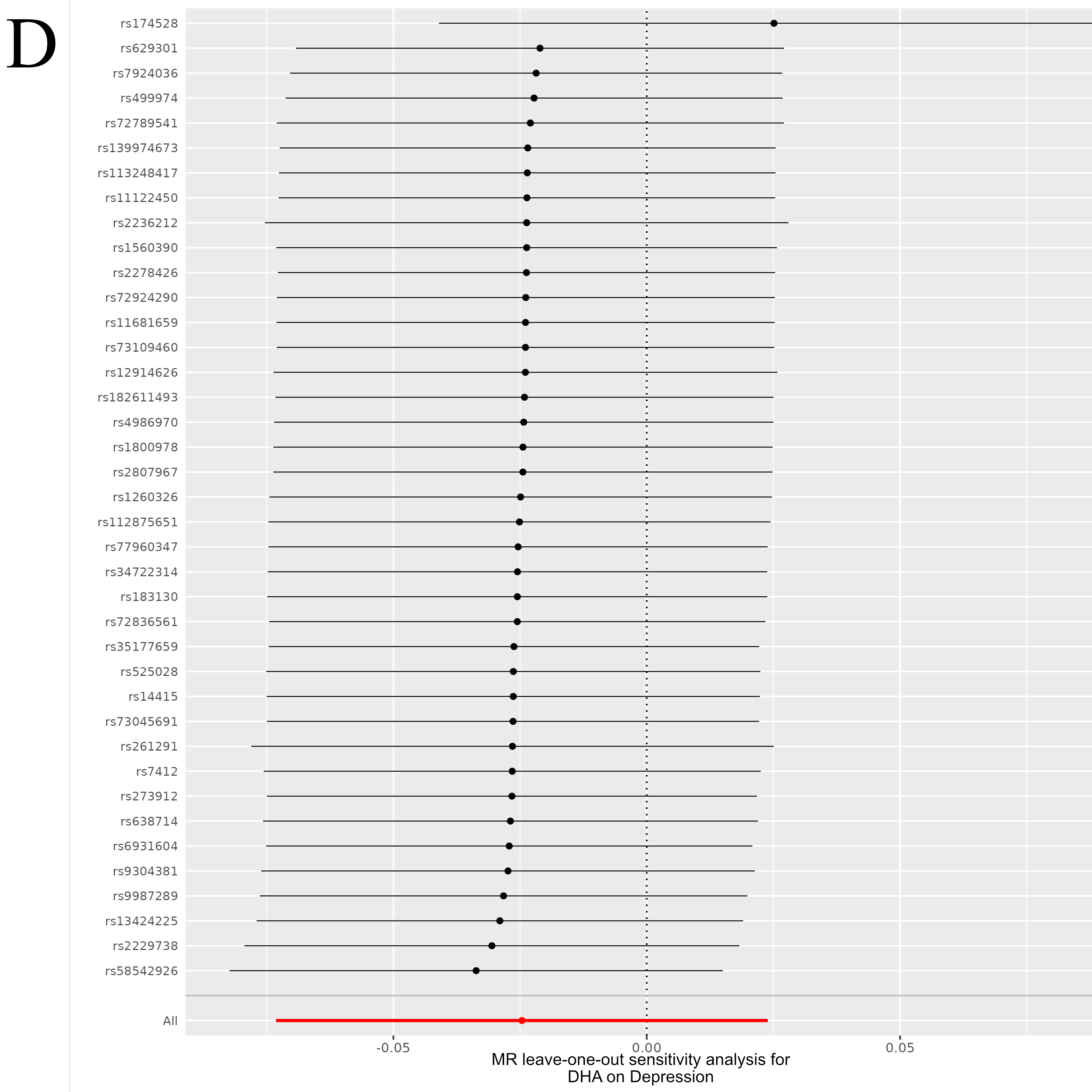
**

**Figure S4. Mendelian randomization (MR) sensitivity plots of the causal effect of Omega-6 Fatty Acids levels on Depression.**

**A. Scatter plot.** Scatter plots of genetic association with **Omega-6 Fatty Acids levels** on Depression showing comparison of the causal estimates from the various Mendelian randomization methods.

**
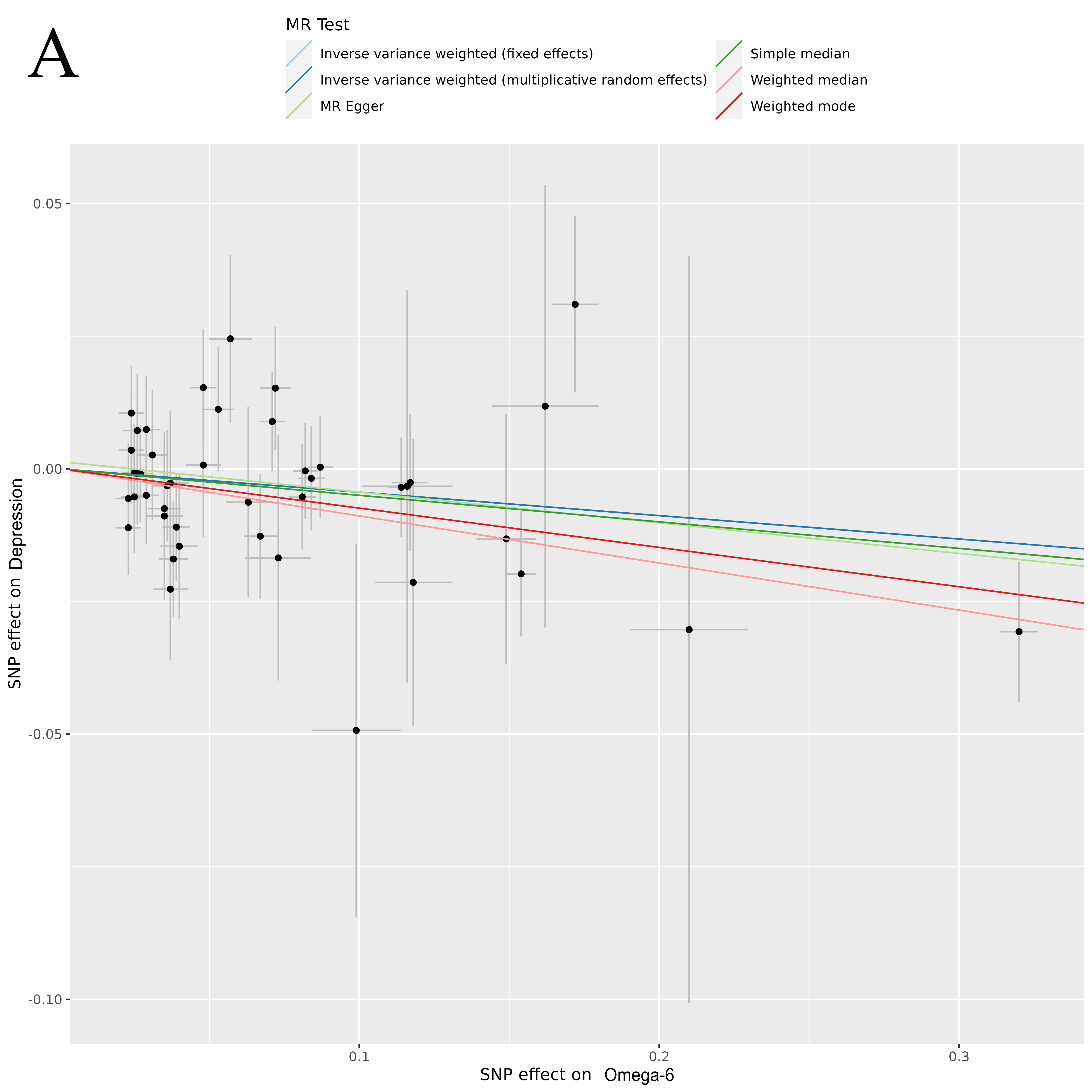
**

**B. Funnel plot.** Funnel plot of causal association between Omega-6 Fatty Acids levels and Depression.**
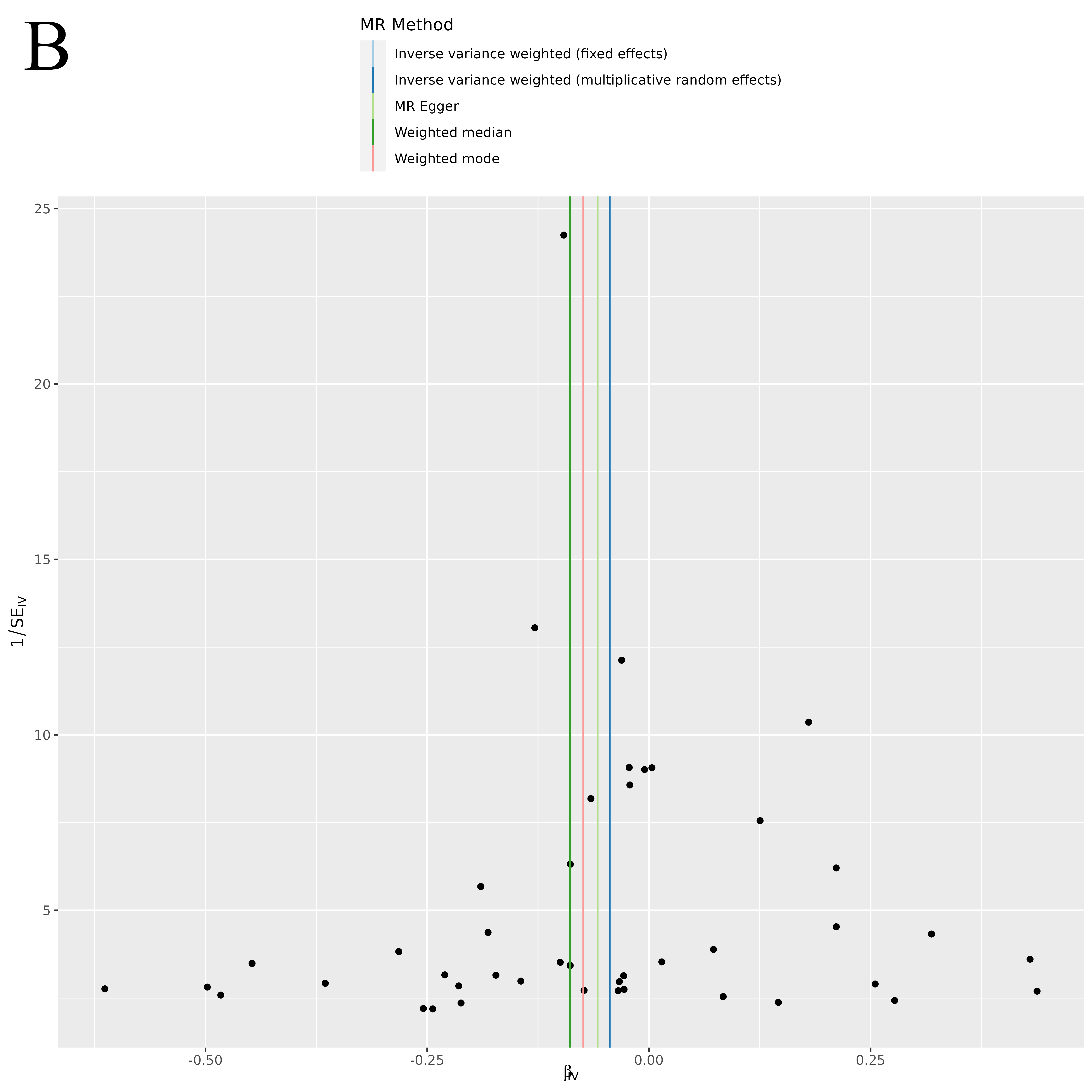
**

**C. Forest plot.** Forest plot of variant specific inverse variance estimates for causal association between Omega-6 Fatty Acids levels and Depression.

**
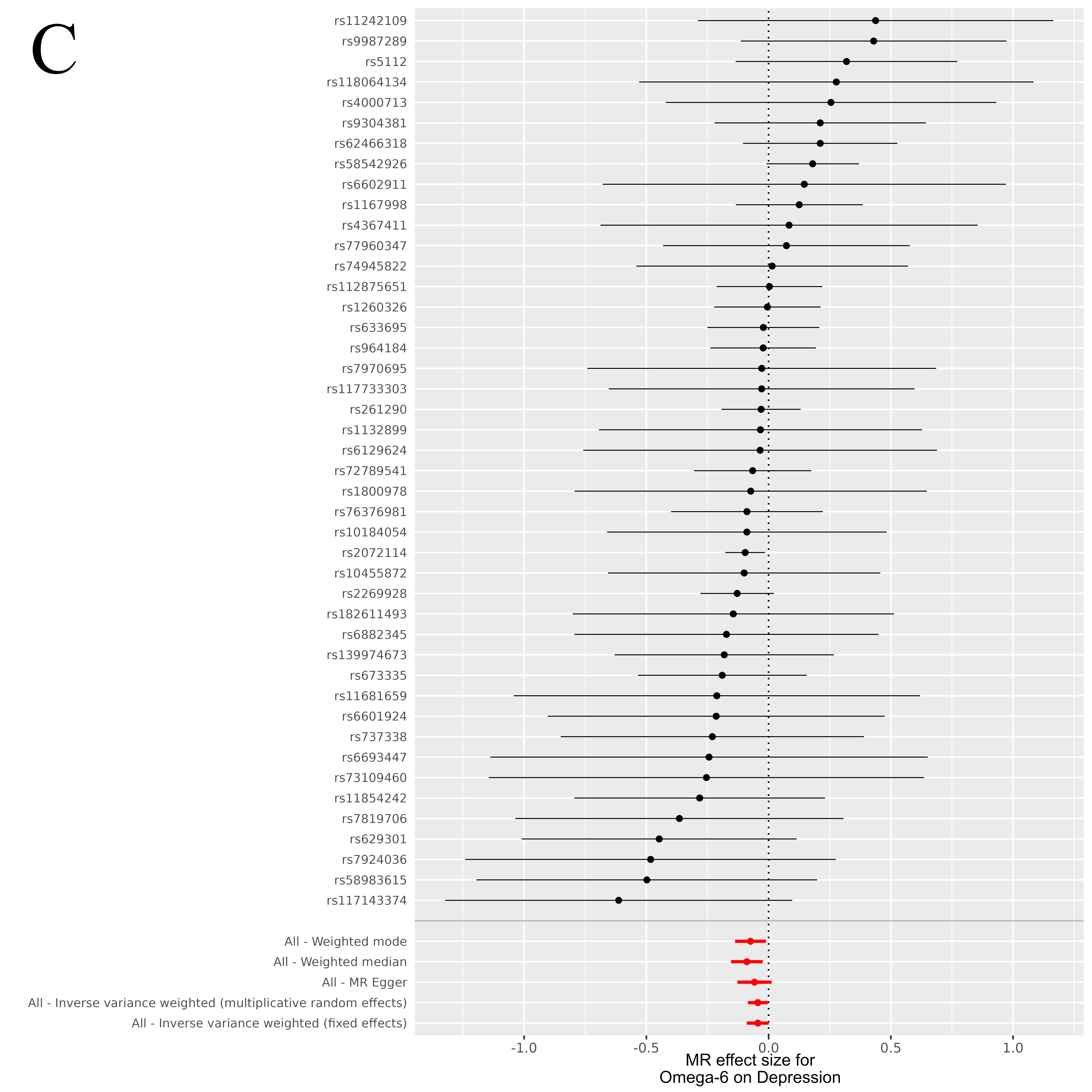
**

**D. Leave-one-out plot.** Leave-one-out plot to assess if a single variant is driving the association between Omega-6 Fatty Acids levels and Depression.

**
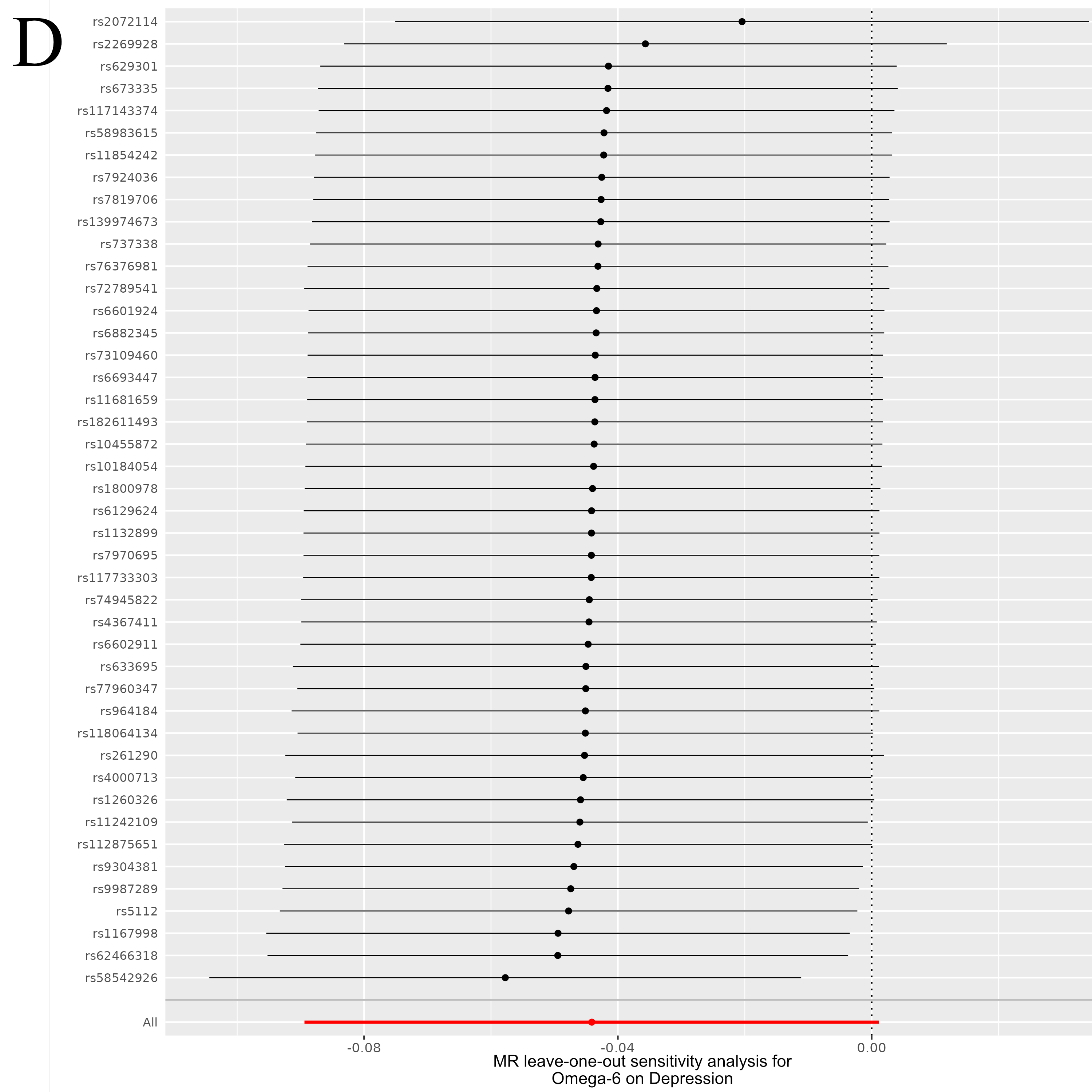
**

**Figure S5. Mendelian randomization (MR) sensitivity plots of the causal effect of Stearic acid (SA) levels on Depression.**

**A. Scatter plot.** Scatter plots of genetic association with Stearic acid (SA) levels on Depression showing comparison of the causal estimates from the various Mendelian randomization methods.

**
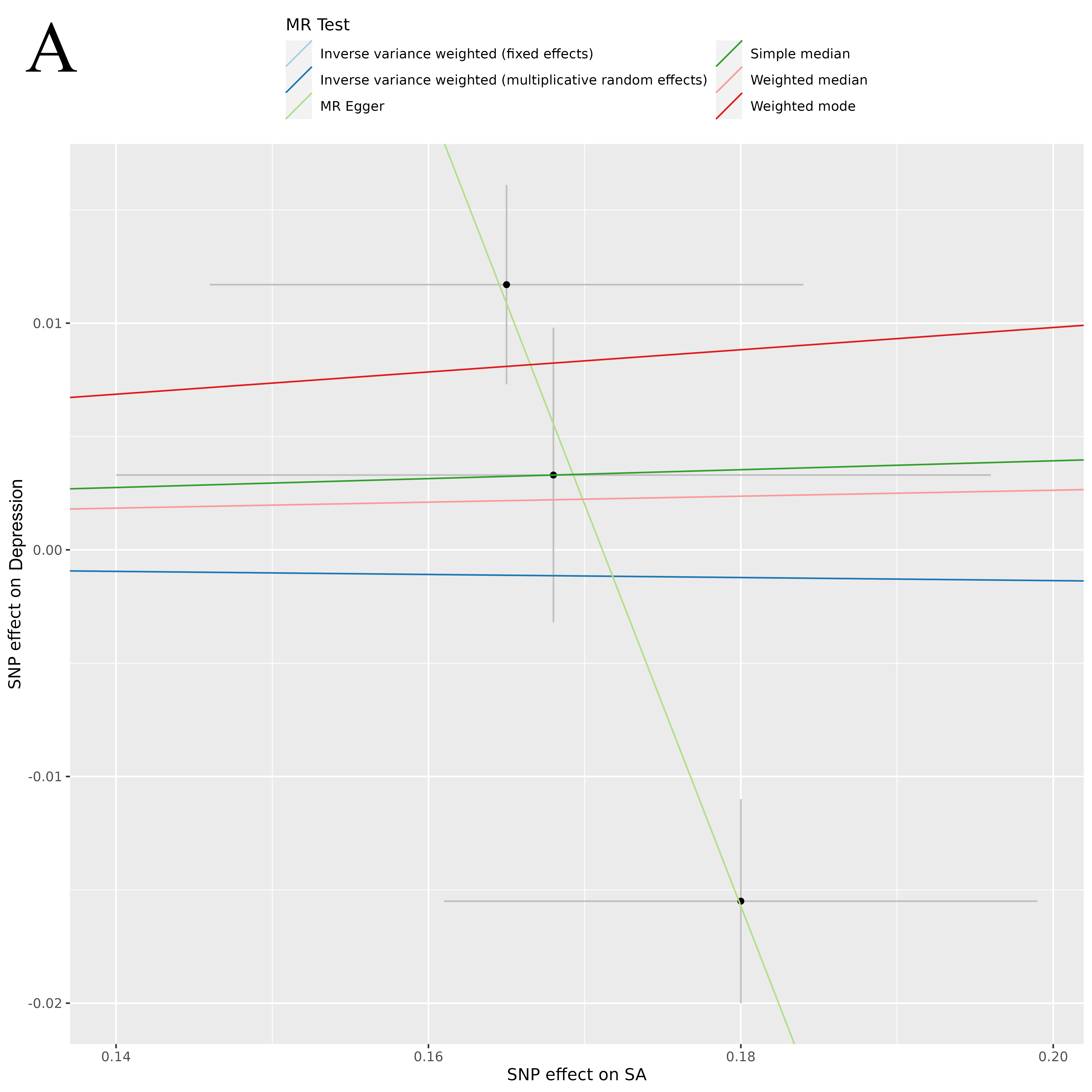
**

**B. Funnel plot.** Funnel plot of causal association between Stearic acid (SA) levels and Depression.**
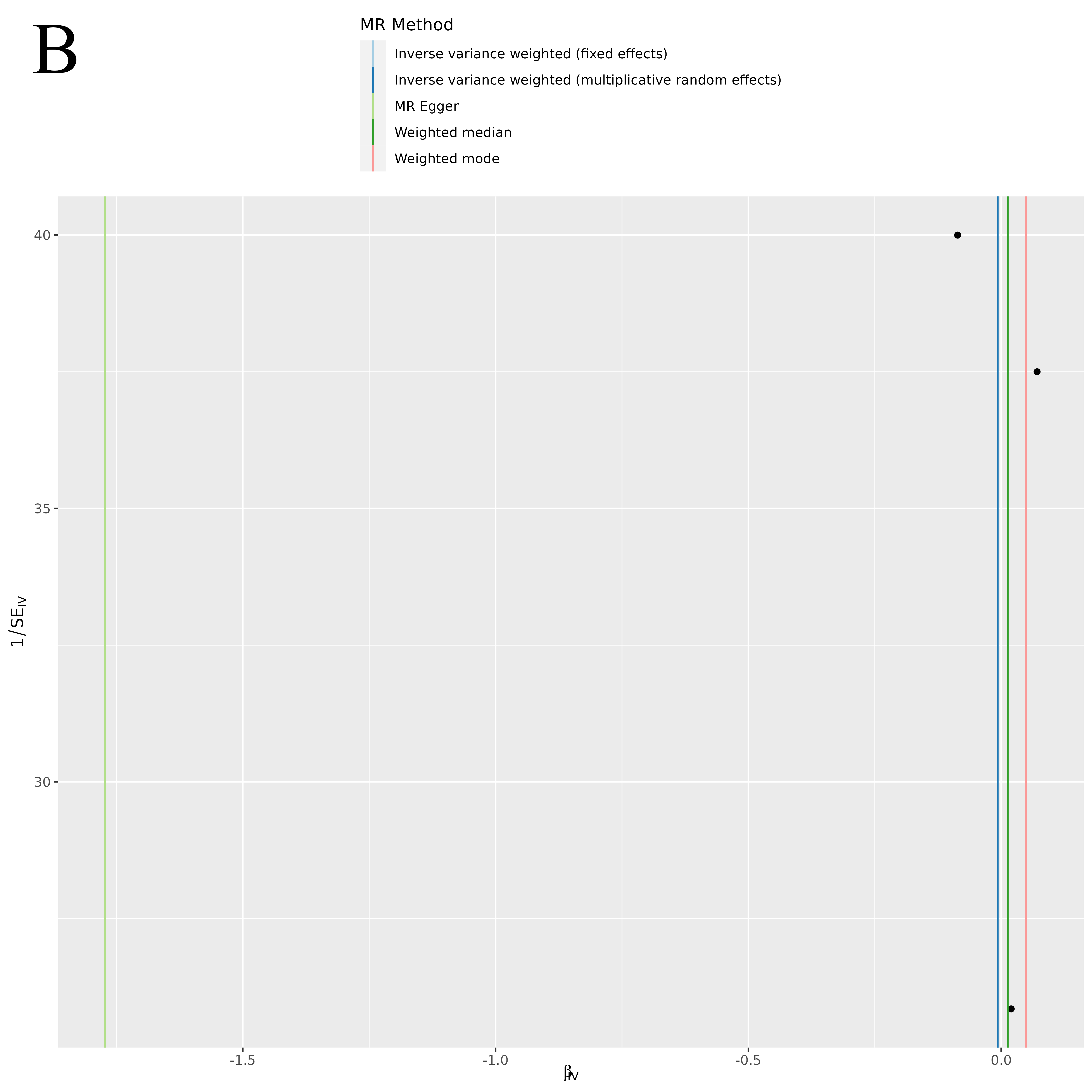
**

**C. Forest plot.** Forest plot of variant specific inverse variance estimates for causal association between Stearic acid (SA) levels and Depression.

**
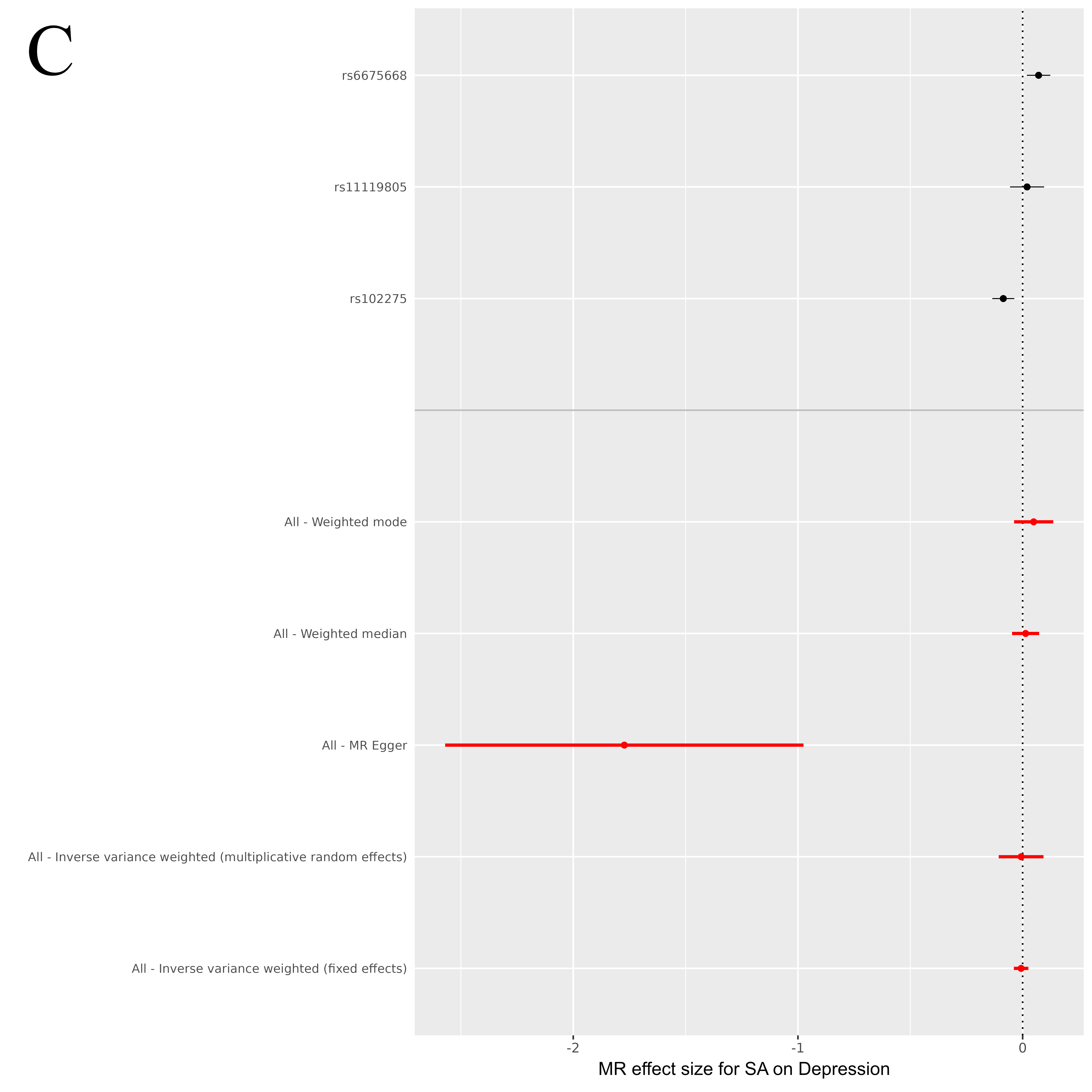
**

**D. Leave-one-out plot.** Leave-one-out plot to assess if a single variant is driving the association between Stearic acid (SA) levels and Depression.

**
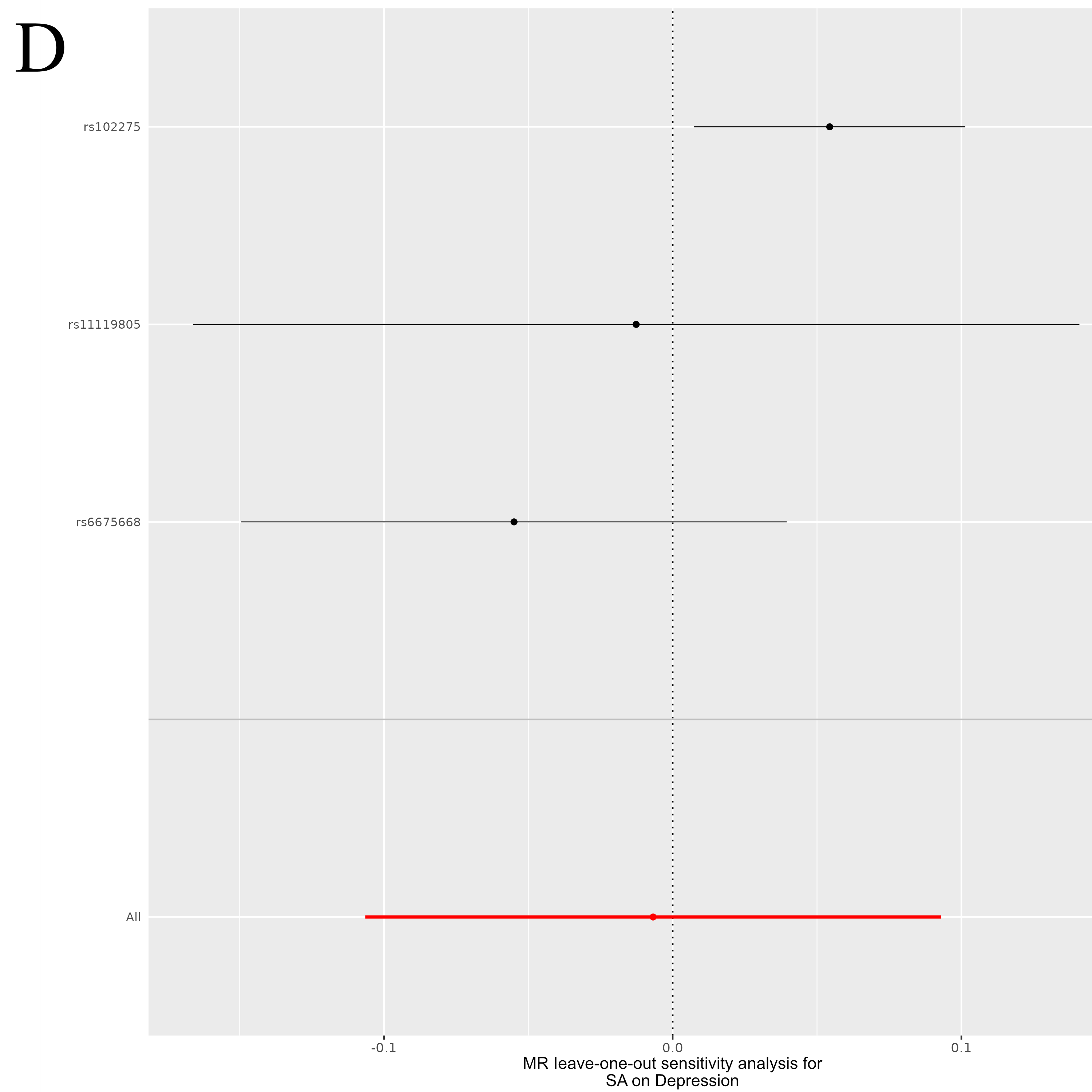
**

**Figure S6. Mendelian randomization (MR) sensitivity plots of the causal effect of Palmitoleic acid (POA) levels on Depression.**

**A. Scatter plot.** Scatter plots of genetic association with Palmitoleic acid (POA) levels on Depression showing comparison of the causal estimates from the various Mendelian randomization methods.

**
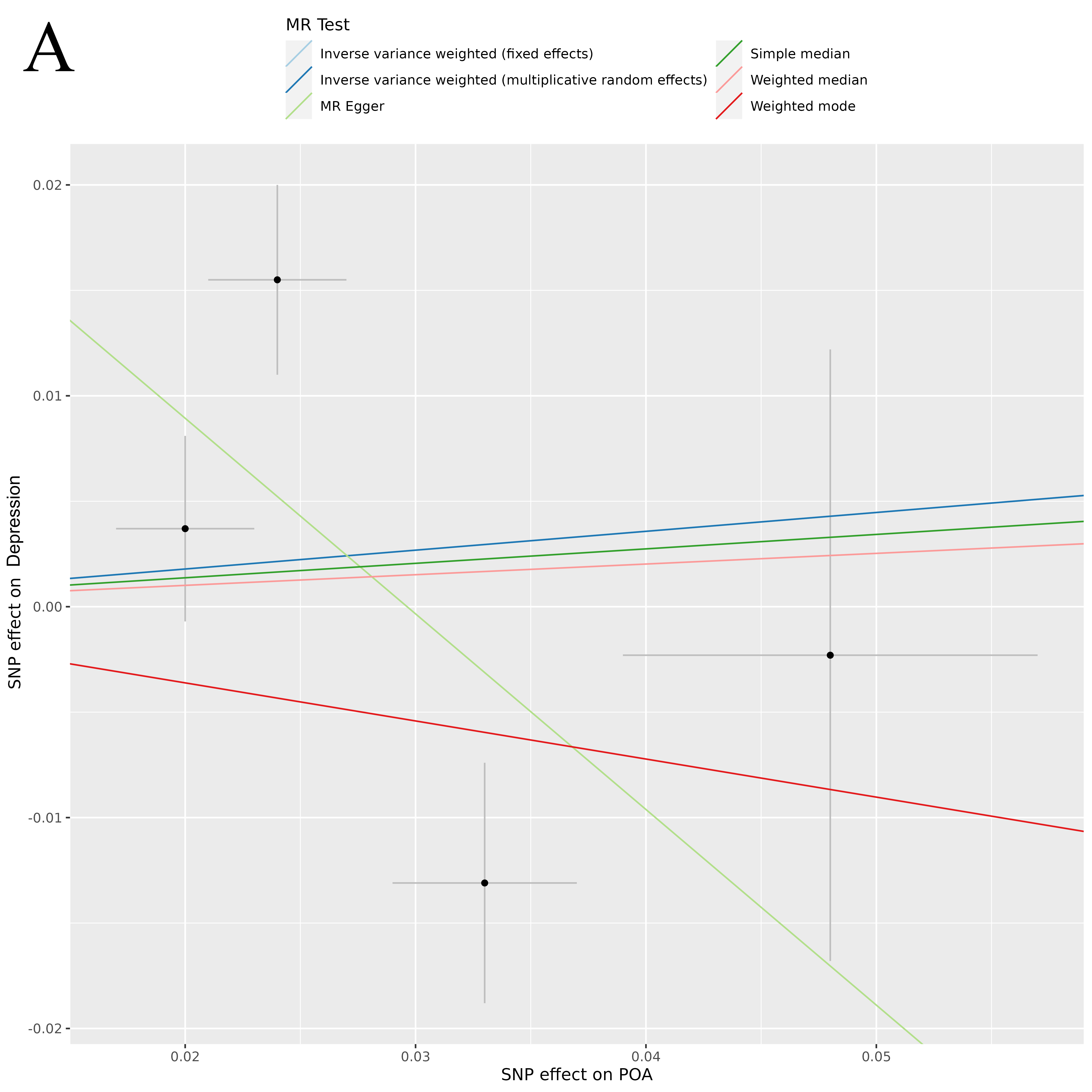
**

**B. Funnel plot.** Funnel plot of causal association between Palmitoleic acid (POA) levels and Depression.**
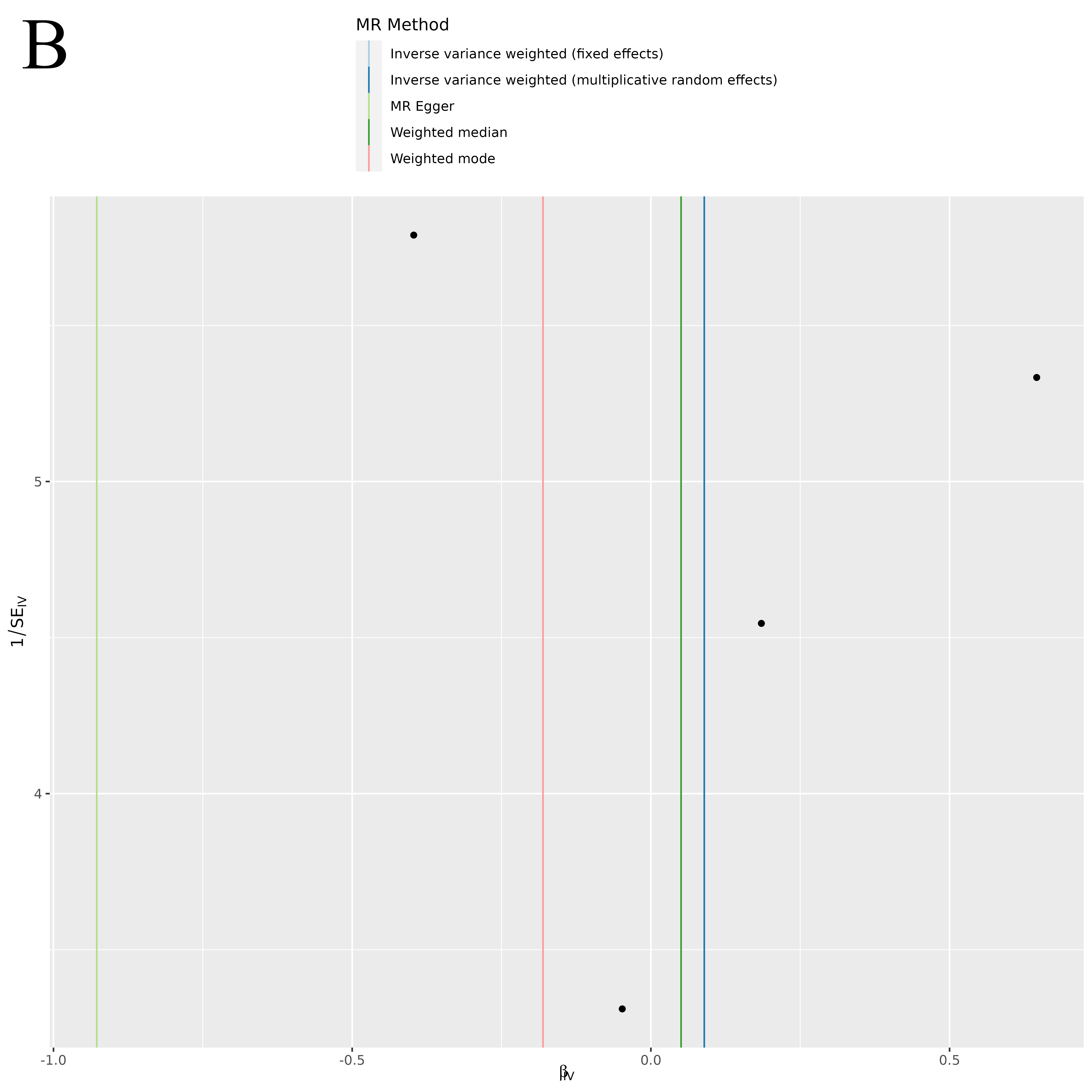
**

**C. Forest plot.** Forest plot of variant specific inverse variance estimates for causal association between Palmitoleic acid (POA) levels and Depression.

**
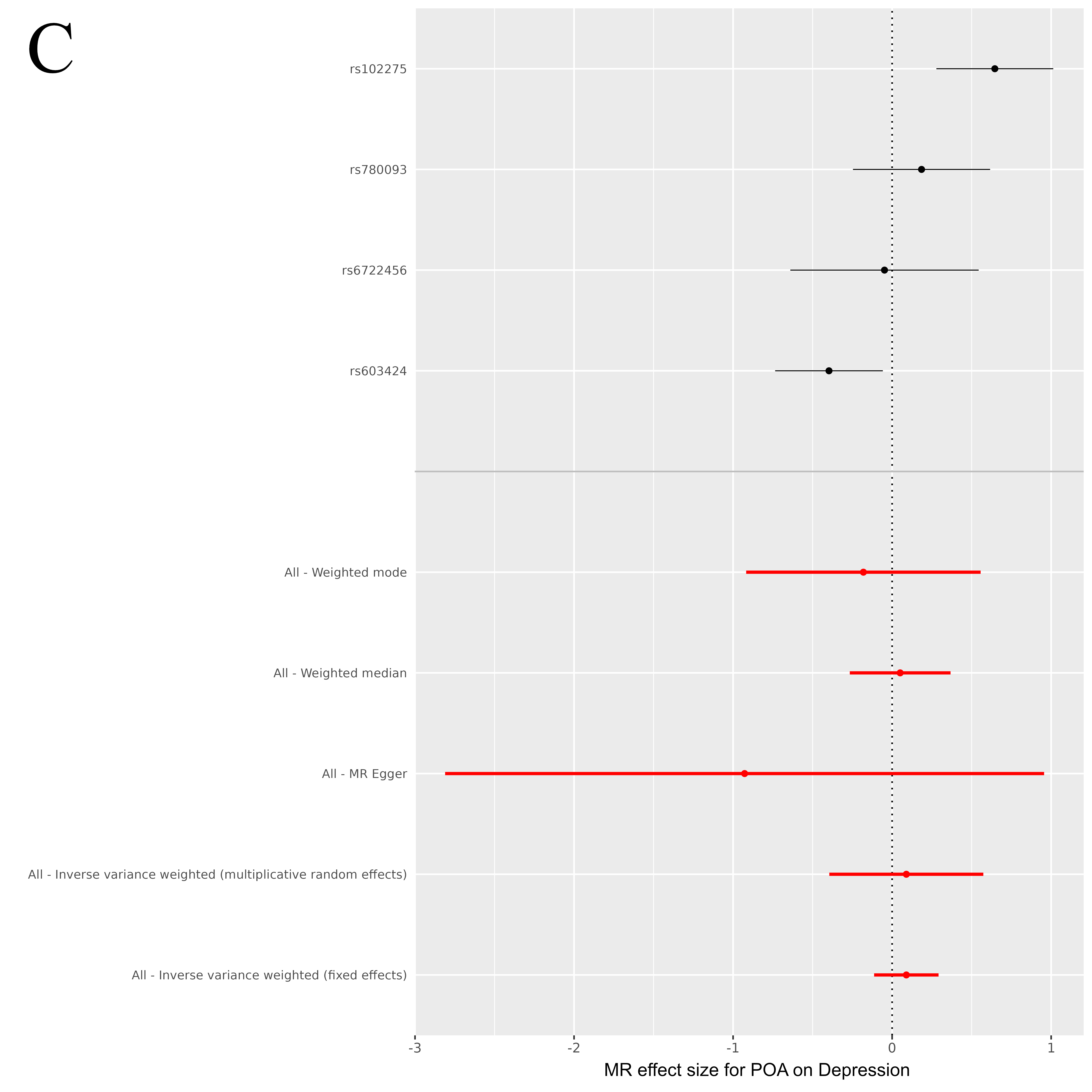
**

**D. Leave-one-out plot.** Leave-one-out plot to assess if a single variant is driving the association between Palmitoleic acid (POA) levels and Depression.

**
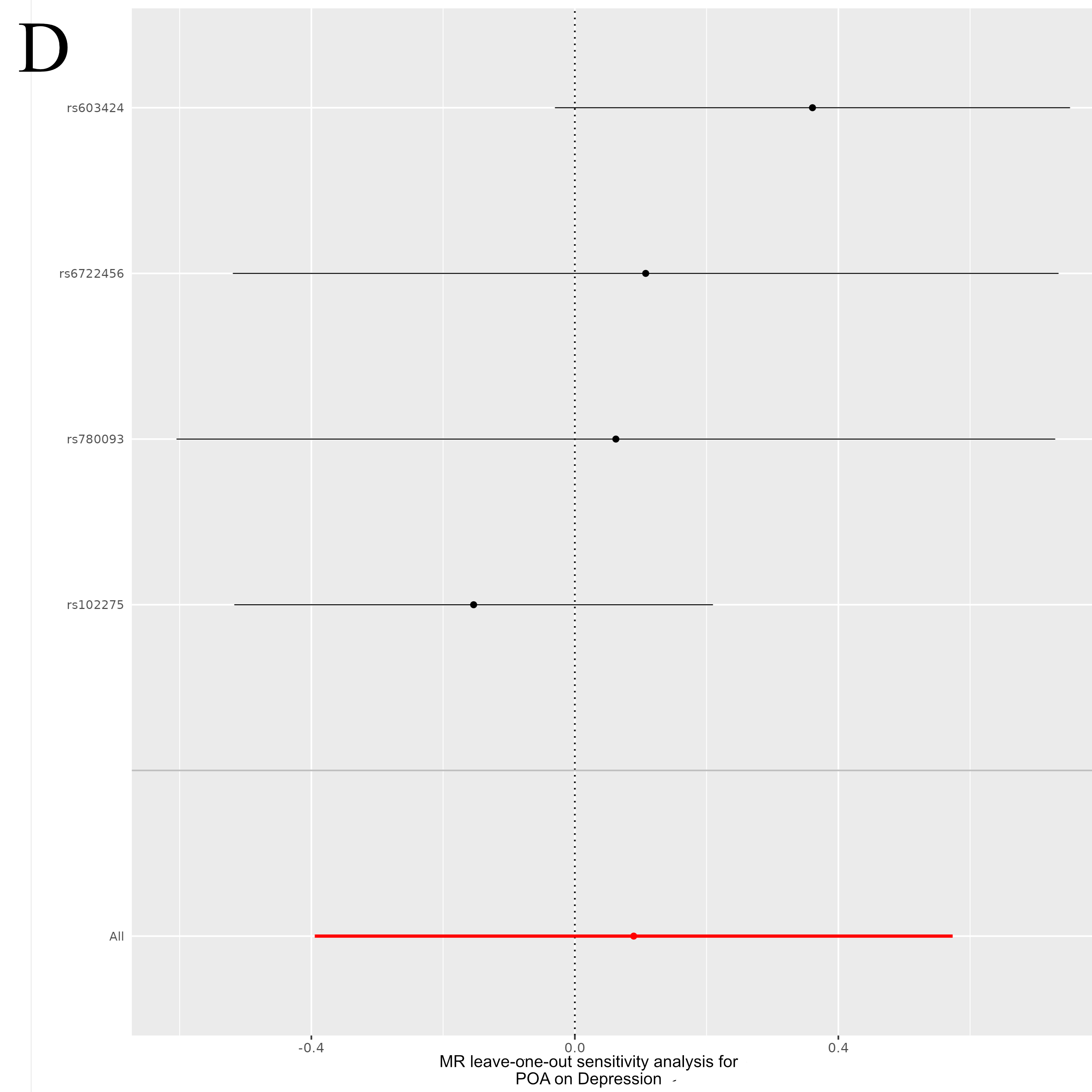
**

# Supplementary Tables

**Table S1. Instrumental variables associated with various subtypes of fatty acids level were used in the present analysis.**

| **Exposure** | **SNP** | **CHR** | **A1** | **A2** | **FRQ** | **BETA** | **SE** | **P** |
| --- | --- | --- | --- | --- | --- | --- | --- | --- |
| AA | rs174547 | 11 | T | C | 0.68 | 1.691 | 0.025 | 1.00E-200 |
| AA | rs16966952 | 16 | G | A | 0.69 | 0.199 | 0.031 | 2.43E-10 |
| Adrenic acid | rs174547 | 11 | T | C | 0.67 | 0.0483 | 0.0019 | 6.26E-140 |
| ALA | rs174547 | 11 | C | T | 0.33 | 0.016 | 0.001 | 3.47E-64 |
| Arachidic acid | rs680379 | 20 | A | G | 0.63 | 0.098 | 0.01 | 5.81E-13 |
| DGLA | rs174547 | 11 | C | T | 0.33 | 0.355 | 0.0136 | 2.63E-151 |
| DGLA | rs16966952 | 16 | G | A | 0.69 | 0.22 | 0.013 | 7.55E-65 |
| DHA | rs2236212 | 6 | G | C | 0.57 | 0.113 | 0.014 | 1.26E-15 |
| DHA | rs638714 | 1 | G | T | 0.65 | 0.038 | 0.0042 | 2.80E-19 |
| DHA | rs629301 | 1 | G | T | 0.22 | -0.044 | 0.0048 | 7.30E-20 |
| DHA | rs11122450 | 1 | T | G | 0.39 | -0.023 | 0.0041 | 3.30E-08 |
| DHA | rs34722314 | 2 | T | A | 0.86 | 0.049 | 0.0059 | 3.20E-17 |
| DHA | rs1260326 | 2 | T | C | 0.4 | 0.047 | 0.0041 | 2.50E-30 |
| DHA | rs11681659 | 2 | C | T | 0.28 | 0.029 | 0.0044 | 2.20E-11 |
| DHA | rs13424225 | 2 | G | T | 0.55 | -0.022 | 0.004 | 2.50E-08 |
| DHA | rs34707604 | 4 | T | C | 0.74 | -0.039 | 0.0048 | 1.20E-15 |
| DHA | rs273912 | 5 | G | T | 0.29 | -0.026 | 0.0044 | 3.80E-09 |
| DHA | rs2394976 | 6 | G | T | 0.84 | 0.034 | 0.0054 | 2.10E-10 |
| DHA | rs6931604 | 6 | C | T | 0.4 | -0.023 | 0.0041 | 2.10E-08 |
| DHA | rs35177659 | 7 | C | T | 0.48 | -0.023 | 0.0042 | 3.40E-08 |
| DHA | rs73109460 | 7 | G | A | 0.88 | 0.037 | 0.0061 | 1.60E-09 |
| DHA | rs14415 | 7 | T | C | 0.72 | 0.029 | 0.0044 | 3.20E-11 |
| DHA | rs9987289 | 8 | A | G | 0.09 | -0.058 | 0.0069 | 4.90E-17 |
| DHA | rs117026536 | 8 | G | T | 0.9 | -0.039 | 0.0067 | 5.00E-09 |
| DHA | rs112875651 | 8 | G | A | 0.61 | 0.049 | 0.0041 | 2.30E-32 |
| DHA | rs1800978 | 9 | C | G | 0.88 | 0.034 | 0.0061 | 3.30E-08 |
| DHA | rs2807967 | 10 | T | C | 0.28 | -0.025 | 0.0045 | 1.70E-08 |
| DHA | rs7924036 | 10 | G | T | 0.5 | -0.036 | 0.004 | 4.10E-19 |
| DHA | rs55891451 | 10 | A | C | 0.8 | -0.032 | 0.005 | 9.50E-11 |
| DHA | rs543711028 | 11 | G | C | 0.99 | 0.178 | 0.0181 | 9.10E-23 |
| DHA | rs174528 | 11 | T | C | 0.62 | 0.265 | 0.0041 | 1.00E-200 |
| DHA | rs72924290 | 11 | T | C | 0.96 | -0.086 | 0.0112 | 1.30E-14 |
| DHA | rs113248417 | 11 | A | G | 0.98 | 0.086 | 0.013 | 4.40E-11 |
| DHA | rs2229738 | 11 | C | T | 0.93 | 0.127 | 0.008 | 4.80E-57 |
| DHA | rs499974 | 11 | C | A | 0.84 | 0.055 | 0.0055 | 5.90E-24 |
| DHA | rs525028 | 11 | G | A | 0.29 | 0.03 | 0.0044 | 1.90E-11 |
| DHA | rs78689694 | 11 | G | C | 0.87 | -0.038 | 0.0059 | 1.10E-10 |
| DHA | rs139974673 | 15 | T | C | 0.97 | -0.071 | 0.0126 | 1.70E-08 |
| DHA | rs1560390 | 15 | T | C | 0.78 | 0.05 | 0.0048 | 4.20E-25 |
| DHA | rs261291 | 15 | T | C | 0.64 | -0.115 | 0.0042 | 1.00E-167 |
| DHA | rs12914626 | 15 | C | T | 0.3 | 0.061 | 0.0044 | 9.90E-44 |
| DHA | rs199782954 | 16 | G | A | 0.72 | -0.04 | 0.0049 | 1.00E-15 |
| DHA | rs72789541 | 16 | T | A | 0.7 | 0.078 | 0.0044 | 1.20E-70 |
| DHA | rs183130 | 16 | C | T | 0.68 | -0.039 | 0.0043 | 3.60E-20 |
| DHA | rs4986970 | 16 | A | T | 0.97 | 0.069 | 0.0111 | 4.40E-10 |
| DHA | rs72836561 | 17 | C | T | 0.97 | 0.063 | 0.0114 | 3.30E-08 |
| DHA | rs16940904 | 17 | C | T | 0.77 | 0.04 | 0.0048 | 1.50E-16 |
| DHA | rs77960347 | 18 | A | G | 0.99 | -0.142 | 0.0174 | 4.10E-16 |
| DHA | rs9304381 | 18 | C | T | 0.18 | -0.05 | 0.0052 | 2.30E-22 |
| DHA | rs2278426 | 19 | C | T | 0.96 | 0.06 | 0.0108 | 2.60E-08 |
| DHA | rs58542926 | 19 | C | T | 0.93 | 0.119 | 0.0076 | 5.90E-55 |
| DHA | rs182611493 | 19 | A | G | 0.99 | 0.157 | 0.0192 | 2.70E-16 |
| DHA | rs7412 | 19 | C | T | 0.92 | 0.077 | 0.0074 | 1.80E-25 |
| DHA | rs73045691 | 19 | G | A | 0.7 | -0.029 | 0.0046 | 1.60E-10 |
| DPA | rs780094 | 2 | T | C | 0.41 | 0.017 | 0.003 | 9.04E-09 |
| DPA | rs3734398 | 6 | C | T | 0.43 | 0.04 | 0.003 | 9.61E-44 |
| DPA | rs174547 | 11 | T | C | 0.67 | 0.075 | 0.003 | 3.79E-154 |
| EPA | rs3798713 | 6 | C | G | 0.43 | 0.035 | 0.005 | 1.93E-12 |
| EPA | rs174538 | 11 | G | A | 0.72 | 0.083 | 0.005 | 5.37E-58 |
| GLA | rs174547 | 11 | T | C | 0.67 | 0.0156 | 9.00E-04 | 2.29E-72 |
| GLA | rs16966952 | 16 | G | A | 0.69 | 0.0061 | 9.00E-04 | 5.05E-11 |
| LA | rs10740118 | 10 | G | C | 0.56 | 0.248 | 0.043 | 8.08E-09 |
| LA | rs174547 | 11 | C | T | 0.32 | 1.474 | 0.042 | 1.00E-200 |
| LA | rs16966952 | 16 | G | A | 0.69 | 0.351 | 0.044 | 1.23E-15 |
| LA | rs534417 | 1 | A | G | 0.12 | -0.036 | 0.0061 | 3.70E-09 |
| LA | rs2986164 | 1 | G | A | 0.46 | 0.027 | 0.0044 | 1.70E-09 |
| LA | rs34232196 | 1 | C | T | 0.75 | 0.031 | 0.0047 | 1.10E-10 |
| LA | rs1002687 | 1 | G | A | 0.36 | -0.087 | 0.0042 | 1.30E-93 |
| LA | rs602633 | 1 | T | G | 0.22 | -0.053 | 0.0049 | 2.10E-27 |
| LA | rs822928 | 1 | A | C | 0.47 | -0.029 | 0.0041 | 7.60E-13 |
| LA | rs35633876 | 2 | G | T | 0.52 | 0.034 | 0.0041 | 2.50E-17 |
| LA | rs693 | 2 | G | A | 0.48 | -0.061 | 0.0041 | 6.40E-52 |
| LA | rs4665972 | 2 | T | C | 0.4 | 0.051 | 0.0042 | 6.90E-35 |
| LA | rs4299376 | 2 | G | T | 0.32 | 0.039 | 0.0043 | 4.30E-19 |
| LA | rs2389599 | 2 | G | C | 0.48 | -0.023 | 0.0041 | 1.60E-08 |
| LA | rs13108218 | 4 | A | G | 0.38 | 0.033 | 0.0042 | 6.30E-15 |
| LA | rs4704210 | 5 | G | C | 0.63 | -0.051 | 0.0042 | 2.60E-34 |
| LA | rs6882345 | 5 | G | A | 0.37 | -0.044 | 0.0042 | 7.50E-26 |
| LA | rs9391844 | 6 | A | G | 0.82 | -0.034 | 0.0053 | 7.70E-11 |
| LA | rs11755689 | 6 | A | G | 0.67 | -0.043 | 0.0044 | 1.40E-22 |
| LA | rs9273453 | 6 | C | G | 0.87 | 0.045 | 0.0065 | 6.30E-12 |
| LA | rs7750288 | 6 | A | G | 0.71 | -0.027 | 0.0045 | 2.90E-09 |
| LA | rs3011437 | 6 | T | G | 0.71 | -0.036 | 0.0045 | 1.30E-15 |
| LA | rs186696265 | 6 | C | T | 0.99 | 0.227 | 0.0171 | 2.60E-40 |
| LA | rs34121855 | 7 | T | G | 0.8 | 0.045 | 0.005 | 3.70E-19 |
| LA | rs1461729 | 8 | A | G | 0.1 | -0.07 | 0.0067 | 1.50E-25 |
| LA | rs7816447 | 8 | T | C | 0.9 | 0.049 | 0.0067 | 4.10E-13 |
| LA | rs6471717 | 8 | G | A | 0.34 | 0.031 | 0.0043 | 6.90E-13 |
| LA | rs112875651 | 8 | G | A | 0.61 | 0.051 | 0.0042 | 6.00E-34 |
| LA | rs4008004 | 9 | C | A | 0.78 | -0.031 | 0.0049 | 2.80E-10 |
| LA | rs11789603 | 9 | C | T | 0.89 | -0.045 | 0.0065 | 3.20E-12 |
| LA | rs2740488 | 9 | A | C | 0.73 | 0.048 | 0.0046 | 8.70E-26 |
| LA | rs115478735 | 9 | A | T | 0.82 | -0.043 | 0.0052 | 2.80E-16 |
| LA | rs11239569 | 10 | C | A | 0.75 | -0.03 | 0.0047 | 2.70E-10 |
| LA | rs3817335 | 11 | T | A | 0.65 | 0.026 | 0.0042 | 5.80E-10 |
| LA | rs964184 | 11 | G | C | 0.13 | 0.146 | 0.006 | 1.80E-131 |
| LA | rs141469619 | 11 | A | G | 0.99 | -0.124 | 0.0214 | 7.10E-09 |
| LA | rs12970 | 11 | G | A | 0.94 | 0.047 | 0.0085 | 3.80E-08 |
| LA | rs4766578 | 12 | T | A | 0.5 | -0.026 | 0.0041 | 3.30E-10 |
| LA | rs11065358 | 12 | T | C | 0.34 | 0.027 | 0.0043 | 2.90E-10 |
| LA | rs865716 | 12 | A | T | 0.5 | 0.024 | 0.0041 | 6.30E-09 |
| LA | rs6602911 | 13 | C | T | 0.64 | -0.024 | 0.0042 | 1.70E-08 |
| LA | rs11854242 | 15 | C | T | 0.72 | 0.036 | 0.0046 | 1.80E-15 |
| LA | rs261290 | 15 | T | C | 0.35 | 0.089 | 0.0043 | 9.20E-97 |
| LA | rs633695 | 15 | A | G | 0.71 | -0.07 | 0.0045 | 8.00E-55 |
| LA | rs247617 | 16 | C | A | 0.68 | -0.051 | 0.0043 | 1.40E-31 |
| LA | rs12948283 | 17 | G | C | 0.7 | -0.026 | 0.0047 | 1.60E-08 |
| LA | rs740516 | 17 | C | G | 0.85 | 0.034 | 0.0057 | 3.20E-09 |
| LA | rs77960347 | 18 | A | G | 0.99 | -0.249 | 0.0177 | 7.50E-45 |
| LA | rs4939883 | 18 | T | C | 0.18 | -0.063 | 0.0053 | 5.10E-33 |
| LA | rs142158911 | 19 | G | A | 0.88 | 0.092 | 0.0063 | 3.20E-47 |
| LA | rs56322906 | 19 | G | A | 0.96 | 0.088 | 0.011 | 1.00E-15 |
| LA | rs58542926 | 19 | C | T | 0.93 | 0.11 | 0.0077 | 6.60E-46 |
| LA | rs1081105 | 19 | A | C | 0.97 | -0.121 | 0.0124 | 2.00E-22 |
| LA | rs1065853 | 19 | G | T | 0.92 | 0.189 | 0.0075 | 4.90E-141 |
| LA | rs79429216 | 19 | G | A | 0.99 | -0.147 | 0.018 | 4.00E-16 |
| LA | rs2378390 | 20 | G | A | 0.86 | 0.032 | 0.0058 | 2.70E-08 |
| LA | rs1883711 | 20 | G | C | 0.97 | -0.09 | 0.0119 | 4.60E-14 |
| OA | rs102275 | 11 | C | T | 0.33 | 0.23 | 0.019 | 2.19E-32 |
| PA | rs2391388 | 1 | C | A | 0.45 | 0.178 | 0.027 | 2.72E-11 |
| POA | rs780093 | 2 | T | C | 0.41 | 0.02 | 0.003 | 9.80E-10 |
| POA | rs6722456 | 2 | G | A | 0.98 | 0.048 | 0.009 | 4.12E-08 |
| POA | rs603424 | 10 | G | A | 0.81 | 0.033 | 0.004 | 5.69E-15 |
| POA | rs102275 | 11 | C | T | 0.33 | 0.024 | 0.003 | 6.60E-13 |
| SA | rs6675668 | 1 | G | T | 0.51 | 0.165 | 0.019 | 2.16E-18 |
| SA | rs11119805 | 1 | T | A | 0.88 | 0.168 | 0.028 | 2.80E-09 |
| SA | rs102275 | 11 | T | C | 0.68 | 0.18 | 0.019 | 1.33E-20 |
| Omega_3 | rs6693447 | 1 | T | G | 0.54 | -0.023 | 0.0041 | 1.80E-08 |
| Omega_3 | rs1167998 | 1 | C | A | 0.36 | -0.071 | 0.0043 | 3.00E-63 |
| Omega_3 | rs629301 | 1 | G | T | 0.22 | -0.038 | 0.0049 | 4.50E-15 |
| Omega_3 | rs10184054 | 2 | C | G | 0.78 | 0.036 | 0.0049 | 1.10E-13 |
| Omega_3 | rs1260326 | 2 | T | C | 0.4 | 0.082 | 0.0042 | 7.00E-87 |
| Omega_3 | rs11681659 | 2 | C | T | 0.28 | 0.025 | 0.0045 | 2.10E-08 |
| Omega_3 | rs34707604 | 4 | T | C | 0.74 | -0.046 | 0.0049 | 5.30E-21 |
| Omega_3 | rs11242109 | 5 | G | T | 0.52 | -0.024 | 0.0041 | 3.30E-09 |
| Omega_3 | rs6882345 | 5 | G | A | 0.37 | -0.029 | 0.0042 | 7.00E-12 |
| Omega_3 | rs2394976 | 6 | G | T | 0.84 | 0.046 | 0.0055 | 5.40E-17 |
| Omega_3 | rs4367411 | 6 | C | T | 0.79 | -0.031 | 0.0053 | 7.20E-09 |
| Omega_3 | rs117733303 | 6 | A | G | 0.98 | 0.116 | 0.0151 | 1.40E-14 |
| Omega_3 | rs10455872 | 6 | A | G | 0.92 | 0.063 | 0.0075 | 6.50E-17 |
| Omega_3 | rs4000713 | 7 | G | A | 0.7 | 0.029 | 0.0045 | 1.00E-10 |
| Omega_3 | rs73109460 | 7 | G | A | 0.88 | 0.035 | 0.0062 | 1.90E-08 |
| Omega_3 | rs62466318 | 7 | C | T | 0.8 | 0.072 | 0.0051 | 4.80E-46 |
| Omega_3 | rs9987289 | 8 | A | G | 0.09 | -0.057 | 0.0071 | 1.10E-15 |
| Omega_3 | rs7819706 | 8 | A | G | 0.88 | 0.04 | 0.0063 | 2.90E-10 |
| Omega_3 | rs112875651 | 8 | G | A | 0.61 | 0.087 | 0.0042 | 3.20E-95 |
| Omega_3 | rs1800978 | 9 | C | G | 0.88 | 0.037 | 0.0062 | 1.70E-09 |
| Omega_3 | rs6601924 | 10 | T | C | 0.15 | -0.035 | 0.0056 | 5.00E-10 |
| Omega_3 | rs7924036 | 10 | G | T | 0.5 | -0.023 | 0.0041 | 9.20E-09 |
| Omega_3 | rs55891451 | 10 | A | C | 0.8 | -0.034 | 0.0051 | 1.70E-11 |
| Omega_3 | rs76376981 | 11 | G | C | 0.95 | 0.149 | 0.0099 | 1.70E-50 |
| Omega_3 | rs2269928 | 11 | T | G | 0.77 | 0.154 | 0.0049 | 1.00E-200 |
| Omega_3 | rs2072114 | 11 | A | G | 0.88 | 0.32 | 0.0062 | 1.00E-200 |
| Omega_3 | rs58983615 | 11 | A | T | 0.98 | 0.099 | 0.0149 | 2.40E-11 |
| Omega_3 | rs74945822 | 11 | G | A | 0.86 | 0.048 | 0.0059 | 3.70E-16 |
| Omega_3 | rs118064134 | 11 | G | A | 0.76 | -0.026 | 0.0048 | 4.70E-08 |
| Omega_3 | rs673335 | 11 | T | C | 0.84 | 0.067 | 0.0055 | 1.20E-33 |
| Omega_3 | rs964184 | 11 | G | C | 0.13 | 0.117 | 0.006 | 3.90E-85 |
| Omega_3 | rs7970695 | 12 | G | A | 0.38 | 0.025 | 0.0042 | 1.60E-09 |
| Omega_3 | rs6602911 | 13 | C | T | 0.64 | -0.024 | 0.0042 | 2.30E-08 |
| Omega_3 | rs139974673 | 15 | T | C | 0.97 | -0.118 | 0.0128 | 3.20E-20 |
| Omega_3 | rs11854242 | 15 | C | T | 0.72 | 0.039 | 0.0046 | 3.00E-17 |
| Omega_3 | rs261290 | 15 | T | C | 0.35 | 0.114 | 0.0043 | 3.30E-157 |
| Omega_3 | rs633695 | 15 | A | G | 0.71 | -0.084 | 0.0045 | 2.40E-78 |
| Omega_3 | rs199782954 | 16 | G | A | 0.72 | -0.039 | 0.005 | 5.20E-15 |
| Omega_3 | rs72789541 | 16 | T | A | 0.7 | 0.081 | 0.0045 | 7.30E-74 |
| Omega_3 | rs16940904 | 17 | C | T | 0.77 | 0.036 | 0.0049 | 3.20E-13 |
| Omega_3 | rs77960347 | 18 | A | G | 0.99 | -0.162 | 0.0178 | 8.30E-20 |
| Omega_3 | rs9304381 | 18 | C | T | 0.18 | -0.053 | 0.0053 | 1.20E-23 |
| Omega_3 | rs737338 | 19 | C | T | 0.96 | 0.073 | 0.011 | 4.60E-11 |
| Omega_3 | rs58542926 | 19 | C | T | 0.93 | 0.172 | 0.0078 | 1.20E-108 |
| Omega_3 | rs182611493 | 19 | A | G | 0.99 | 0.21 | 0.0196 | 9.70E-27 |
| Omega_3 | rs5112 | 19 | C | G | 0.47 | -0.048 | 0.0044 | 1.10E-27 |
| Omega_3 | rs1132899 | 19 | T | C | 0.49 | -0.027 | 0.0041 | 4.00E-11 |
| Omega_3 | rs6129624 | 20 | G | A | 0.66 | 0.026 | 0.0044 | 4.00E-09 |
| Omega_3 | rs117143374 | 21 | T | C | 0.86 | 0.037 | 0.0058 | 2.20E-10 |
| Omega_6 | rs534417 | 1 | A | G | 0.12 | -0.039 | 0.0061 | 1.30E-10 |
| Omega_6 | rs2986164 | 1 | G | A | 0.46 | 0.025 | 0.0044 | 1.40E-08 |
| Omega_6 | rs34232196 | 1 | C | T | 0.75 | 0.032 | 0.0047 | 1.20E-11 |
| Omega_6 | rs1002687 | 1 | G | A | 0.36 | -0.091 | 0.0042 | 2.10E-103 |
| Omega_6 | rs660240 | 1 | T | C | 0.22 | -0.058 | 0.0049 | 3.40E-32 |
| Omega_6 | rs496654 | 1 | A | C | 0.48 | -0.03 | 0.004 | 4.60E-14 |
| Omega_6 | rs870526 | 2 | C | T | 0.48 | 0.032 | 0.004 | 2.90E-15 |
| Omega_6 | rs6547409 | 2 | C | T | 0.95 | 0.081 | 0.0092 | 1.40E-18 |
| Omega_6 | rs672889 | 2 | T | G | 0.14 | -0.076 | 0.0058 | 1.50E-39 |
| Omega_6 | rs1260326 | 2 | T | C | 0.4 | 0.064 | 0.0041 | 8.70E-55 |
| Omega_6 | rs4299376 | 2 | G | T | 0.32 | 0.035 | 0.0043 | 2.60E-16 |
| Omega_6 | rs3770586 | 2 | C | T | 0.52 | 0.023 | 0.0041 | 1.40E-08 |
| Omega_6 | rs13108218 | 4 | A | G | 0.38 | 0.035 | 0.0042 | 3.70E-17 |
| Omega_6 | rs4860948 | 4 | T | A | 0.76 | -0.028 | 0.0047 | 3.10E-09 |
| Omega_6 | rs4704210 | 5 | G | C | 0.63 | -0.047 | 0.0042 | 3.60E-29 |
| Omega_6 | rs6882345 | 5 | G | A | 0.37 | -0.045 | 0.0042 | 6.70E-27 |
| Omega_6 | rs9391844 | 6 | A | G | 0.82 | -0.036 | 0.0053 | 7.20E-12 |
| Omega_6 | rs11755689 | 6 | A | G | 0.67 | -0.042 | 0.0044 | 3.00E-21 |
| Omega_6 | rs9273453 | 6 | C | G | 0.87 | 0.043 | 0.0065 | 2.80E-11 |
| Omega_6 | rs6934962 | 6 | C | T | 0.6 | -0.023 | 0.0041 | 2.80E-08 |
| Omega_6 | rs80254170 | 6 | A | G | 0.92 | -0.042 | 0.0076 | 4.60E-08 |
| Omega_6 | rs662138 | 6 | C | G | 0.81 | -0.03 | 0.0052 | 1.10E-08 |
| Omega_6 | rs3011437 | 6 | T | G | 0.71 | -0.032 | 0.0045 | 4.80E-13 |
| Omega_6 | rs186696265 | 6 | C | T | 0.99 | 0.21 | 0.017 | 5.30E-35 |
| Omega_6 | rs34121855 | 7 | T | G | 0.8 | 0.05 | 0.005 | 5.20E-23 |
| Omega_6 | rs2126259 | 8 | T | C | 0.1 | -0.083 | 0.0067 | 1.20E-35 |
| Omega_6 | rs6471717 | 8 | G | A | 0.34 | 0.029 | 0.0043 | 1.20E-11 |
| Omega_6 | rs2721961 | 8 | T | G | 0.72 | 0.027 | 0.0045 | 1.00E-09 |
| Omega_6 | rs112875651 | 8 | G | A | 0.61 | 0.064 | 0.0042 | 3.20E-52 |
| Omega_6 | rs7831074 | 8 | C | G | 0.24 | -0.028 | 0.005 | 3.50E-08 |
| Omega_6 | rs4008004 | 9 | C | A | 0.78 | -0.033 | 0.0049 | 1.40E-11 |
| Omega_6 | rs11789603 | 9 | C | T | 0.89 | -0.048 | 0.0065 | 1.60E-13 |
| Omega_6 | rs2740488 | 9 | A | C | 0.73 | 0.05 | 0.0046 | 6.80E-28 |
| Omega_6 | rs115478735 | 9 | A | T | 0.82 | -0.042 | 0.0052 | 5.30E-16 |
| Omega_6 | rs117488242 | 10 | A | G | 0.87 | 0.035 | 0.0063 | 1.80E-08 |
| Omega_6 | rs11239569 | 10 | C | A | 0.75 | -0.029 | 0.0047 | 3.60E-10 |
| Omega_6 | rs3817335 | 11 | T | A | 0.65 | 0.028 | 0.0042 | 4.50E-11 |
| Omega_6 | rs72997616 | 11 | C | A | 0.91 | 0.052 | 0.0069 | 1.10E-13 |
| Omega_6 | rs964184 | 11 | G | C | 0.13 | 0.139 | 0.0059 | 9.50E-121 |
| Omega_6 | rs4766578 | 12 | T | A | 0.5 | -0.028 | 0.004 | 6.20E-12 |
| Omega_6 | rs7970695 | 12 | G | A | 0.38 | 0.03 | 0.0042 | 4.00E-13 |
| Omega_6 | rs6602911 | 13 | C | T | 0.64 | -0.026 | 0.0042 | 8.80E-10 |
| Omega_6 | rs11854242 | 15 | C | T | 0.72 | 0.038 | 0.0045 | 2.90E-17 |
| Omega_6 | rs261290 | 15 | T | C | 0.35 | 0.097 | 0.0043 | 3.40E-114 |
| Omega_6 | rs633695 | 15 | A | G | 0.71 | -0.073 | 0.0045 | 1.10E-59 |
| Omega_6 | rs3764261 | 16 | C | A | 0.68 | -0.062 | 0.0043 | 2.30E-46 |
| Omega_6 | rs76116020 | 16 | A | G | 0.96 | 0.055 | 0.0099 | 2.60E-08 |
| Omega_6 | rs4561509 | 17 | G | A | 0.5 | -0.022 | 0.004 | 2.90E-08 |
| Omega_6 | rs740516 | 17 | C | G | 0.85 | 0.032 | 0.0057 | 2.40E-08 |
| Omega_6 | rs77960347 | 18 | A | G | 0.99 | -0.276 | 0.0176 | 3.80E-55 |
| Omega_6 | rs9304381 | 18 | C | T | 0.18 | -0.07 | 0.0052 | 5.10E-41 |
| Omega_6 | rs142158911 | 19 | G | A | 0.88 | 0.094 | 0.0063 | 1.80E-50 |
| Omega_6 | rs56322906 | 19 | G | A | 0.96 | 0.1 | 0.011 | 5.30E-20 |
| Omega_6 | rs58542926 | 19 | C | T | 0.93 | 0.128 | 0.0077 | 2.70E-62 |
| Omega_6 | rs74747585 | 19 | T | C | 0.97 | 0.073 | 0.0132 | 3.10E-08 |
| Omega_6 | rs111278137 | 19 | G | A | 0.98 | 0.079 | 0.0144 | 4.10E-08 |
| Omega_6 | rs1081105 | 19 | A | C | 0.97 | -0.119 | 0.0123 | 5.90E-22 |
| Omega_6 | rs1065853 | 19 | G | T | 0.92 | 0.199 | 0.0074 | 4.10E-157 |
| Omega_6 | rs79429216 | 19 | G | A | 0.99 | -0.151 | 0.018 | 4.60E-17 |
| Omega_6 | rs2378390 | 20 | G | A | 0.86 | 0.033 | 0.0058 | 1.00E-08 |
| Omega_6 | rs1883711 | 20 | G | C | 0.97 | -0.092 | 0.0119 | 7.10E-15 |
| Omega_6 | rs1800961 | 20 | C | T | 0.97 | 0.074 | 0.0118 | 2.60E-10 |
| Omega_6 | rs5754102 | 22 | C | A | 0.82 | 0.032 | 0.0053 | 2.10E-09 |
| Omega_6 | rs9616847 | 22 | A | T | 0.61 | -0.024 | 0.0042 | 1.00E-08 |

Abbreviations: Chr = Chromosome; EAF = Effect allele frequency; SE = Standard error; SNP = Single-nucleotide polymorphism.

**Table S2. The heterogeneity and pleiotropy test between specific FAs and depression.**

| **Fatty Acids** | **Method** | **Q-statistics** | **P _heterogeneity_** | **Intercept** | **P _pleiotropy_** |  |  |
| --- | --- | --- | --- | --- | --- | --- | --- |
| Stearic acid (18:0) SA | Inverse variance weighted | 18.45 | 1.74E-05 |  |  |  |  |
| Palmitoleie acid (16:1n-7) POA | Inverse variance weighted | 17.13 | 6.64E-04 | 2.74E-02 | 3.88E-01 |  |  |
|  | MR Egger | 10.73 | 4.69E-03 |  |  |  |  |
| Arachidonic acid (20:4n6) AA | Inverse variance weighted | 6.29 | 1.22E-02 |  |  |  |  |
| Gamma-linolenic acid GLA | Inverse variance weighted | 2.42 | 1.20E-01 |  |  |  |  |
| Dihomo-gamma-linolenic acid DGLA | Inverse variance weighted | 17.36 | 3.09E-05 |  |  |  |  |
| Linoleic acid (18:2n6) LA | Inverse variance weighted | 55.09 | 4.53E-02 | -5.40E-04 | 6.22E-01 |  |  |
|  | MR Egger | 54.74 | 3.85E-02 |  |  |  |  |
| Docosapentaenoic acid (22:5n3) DPA | Inverse variance weighted | 4.88 | 8.71E-02 | 1.10E-02 | 2.87E-01 |  |  |
|  | MR Egger | 0.62 | 4.29E-01 |  |  |  |  |
| Docosahexaenoic acid (22:6n3) DHA | Inverse variance weighted | 34.32 | 2.28E-01 | 3.00E-03 | 1.70E-02 |  |  |
|  | MR Egger | 27.91 | 4.69E-01 |  |  |  |  |
| omega 6 | Inverse variance weighted | 50.06 | 3.15E-01 | -7.56E-03 | 4.16E-02 |  |  |
|  | MR Egger | 45.60 | 4.47E-01 |  |  |  |  |
| omega 3 | Inverse variance weighted | 30.51 | 7.27E-01 | 9.86E-04 | 7.30E-01 |  |  |
|  | MR Egger | 30.39 | 6.90E-01 |  |  | |  |

CI, confidence interval; Ph, P-value for heterogeneity; SNP, single nucleotide polymorphism.
